# Large‐scale connectivity, cryptic population structure, and relatedness in Eastern Pacific Olive ridley sea turtles (*Lepidochelys olivacea*)

**DOI:** 10.1002/ece3.6564

**Published:** 2020-07-19

**Authors:** Ian Silver‐Gorges, Julianne Koval, Clara J. Rodriguez‐Zarate, Frank V. Paladino, Mark Jordan

**Affiliations:** ^1^ Department of Biology Center for Marine Conservation and Biology Purdue University‐Fort Wayne Fort Wayne IN USA; ^2^ Marine Turtle Conservation Programme Emirates Nature in Association with World Wide Fund for Nature (WWF) Dubai United Arab Emirates

**Keywords:** bottleneck, conservation genetics, haplotypes, kin, marine connectivity, ordination, sea turtle

## Abstract

Endangered species are grouped into genetically discrete populations to direct conservation efforts. Mitochondrial control region (mtCR) haplotypes are used to elucidate deep divergences between populations, as compared to nuclear microsatellites that can detect recent structuring. When prior populations are unknown, it is useful to subject microsatellite data to clustering and/or ordination population inference. Olive ridley sea turtles (*Lepidochelys olivacea*) are the most abundant sea turtle, yet few studies have characterized olive ridley population structure. Recently, clustering results of olive ridleys in the Eastern Tropical Pacific Ocean suggested weak structuring (*F*_ST_ = 0.02) between Mexico and Central America. We analyzed mtCR haplotypes, new microsatellite genotypes from Costa Rica, and preexisting microsatellite genotypes from olive ridleys across the Eastern Tropical Pacific, to further explore population structuring in this region. We subjected inferred populations to multiple analyses to explore the mechanisms behind their structuring. We found 10 mtCR haplotypes from 60 turtles nesting at three sites in Costa Rica, but did not detect divergence between Costa Rican sites, or between Central America and Mexico. In Costa Rica, clustering suggested one population with no structuring, but ordination suggested four cryptic clusters with moderate structuring (*F*_ST_ = 0.08, *p* < .001). Across the Eastern Tropical Pacific, ordination suggested nine cryptic clusters with moderate structuring (*F*_ST_ = 0.103, *p* < .001) that largely corresponded to Mexican and Central American populations. All ordination clusters displayed significant internal relatedness relative to global relatedness (*p* < .001) and contained numerous sibling pairs. This suggests that broadly dispersed family lineages have proliferated in Eastern Tropical Pacific olive ridleys and corroborates previous work showing basin‐wide connectivity and shallow population structure in this region. The existence of broadly dispersed kin in Eastern Tropical Pacific olive ridleys has implications for management of olive ridleys in this region, and adds to our understanding of sea turtle ecology and life history, particularly in light of the natal‐homing paradigm.

## Graphical abstract

Olive ridley sea turtles (*Lepidochelys olivacea*) in the Eastern Tropical Pacific comprise broadly distributed family lineages. Evolutionary drivers may motivate related individuals to disperse vast distances from natal beaches and contribute to the persistence of family lineages. This dispersal is an interesting exception to the natal‐homing paradigm for sea turtles.

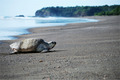

## 1 INTRODUCTION

Understanding the population genetics of endangered species is critical to identifying where and how many distinguishable populations there may be in a region, thus aiding in developing conservation plans for those populations. For sea turtle conservation, this is often done by designating management units (MUs), which are genetically discrete groupings of nesting assemblages (Komoroske, Jensen, Stewart, Shamblin, & Dutton, 2017). Nesting assemblages are obvious choices for defining turtle populations, as females are easily accessible for sampling as they come ashore to nest, and typically display natal homing (Lohmann, Putman, & Lohmann, 2008; Lohmann, Witherington, Lohmann, & Salmon, 2017). Defining MUs is important for developing effective conservation plans and is continually highlighted as a priority for global sea turtle research (Hamann et al., 2010; Rees et al., 2016).

Genetic analyses of mitochondrial DNA and nuclear microsatellites have allowed researchers to designate more informative MUs that capture much of the genetic variation within a species regionally and globally (Bowen & Karl, 2007; Komoroske et al., 2017). Mitochondrial control region (mtCR) sequences (haplotypes) are maternally inherited, and sea turtles typically share haplotypes within regions (such as isolated islands or the northern and southern areas of an ocean basin; Bowen & Karl, 2007), due to maternal natal homing for reproduction. Microsatellite loci are highly variable repeating units found throughout the nuclear genome that may provide novel insights into population structure relative to mtCR haplotypes due to their high mutation rates and biparental heredity. Microsatellites reflect more contemporary gene flow and demographic changes than mtCR haplotypes due to their high mutation rates, and may further be used to conduct analyses to better understand the mechanisms behind MU population structuring (see Blouin, 2003; Putman & Carbone, 2014 for reviews of these analyses).

When populations are unknown, exploratory methods may be used to identify genetically discrete populations. Software that implements clustering population inference (i.e., STRUCTURE; Pritchard, Stephens,& Donnelly, 2000) and ordination population inference (i.e., DAPC; Jombart, Devillard, & Balloux, 2010) is commonly used toward this end. Software that implements clustering population inference typically uses a Bayesian or maximum‐likelihood framework to cluster individuals into arbitrary populations, and then subsequently assesses the likelihoods of those populations and their genetic signatures. Software that implements ordination population inference plots individuals as points on a coordinate plane, and then uses variance between points or groups of points to identify putative populations. These methods have known shortcomings, differ in the assumptions they make of sample data and of the best inferred populations, and may suggest different population structuring when analyzing identical data (see Jombart et al., 2010; Kalinowski, 2011, and Putman & Carbone, 2014). It is therefore critical to use multiple analytical methods when making inferences about population structure in a data set. When studying threatened species such as sea turtles, such thorough analysis will enable researchers and managers to identify population structuring at multiple scales and determine the most suitable MUs for effective conservation and management[Table ece36564-tbl-0001].

**TABLE 1 ece36564-tbl-0001:** Site abbreviations and full names. Table largely adopted from Rodríguez‐Zárate et al. (2018) and Table S2

Abbreviation	Full Site	Study	Population
BCS	Baja California del Sur, Mexico	R‐Z	Mexico
EVE	El Verde, Mexico	R‐Z	Mexico
PLA	Platanitos, Mexico	R‐Z	Mexico
NVA	Nuevo Vallarta, Mexico	R‐Z	Mexico
PVG	Puerto Vallarta/La Gloria, Mexico	R‐Z	Mexico
MIS	Mismaloya, Mexico	R‐Z	Mexico
PTI	Ticuiz, Mexico	R‐Z	Mexico
BAP	Boca de Apiza, Mexico	R‐Z	Mexico
TCO	Tierra Colorada, Mexico	R‐Z	Mexico
SJC	San Juan de Chacahua, Mexico	R‐Z	Mexico
BCR	Barra de la Cruz, Mexico	R‐Z	Mexico
ESC	Escobilla, Mexico	R‐Z	Mexico
PAR	Puerto Arista, Mexico	R‐Z	Mexico
GH	Parque el Hawaii, Guatemala	R‐Z	Central America
SPD	Playa Dorada, El Salvador	R‐Z	Central America
SJG	San Juan del Gozo, El Salvador	R‐Z	Central America
SB	Las Bocanitas/ San Diego, El Salvador	R‐Z	Central America
NC	Chacocente, Nicaragua	R‐Z	Central America
NF	La Flor, Nicaragua	R‐Z	Central America
NV	Playa Veracruz, Nicaragua	R‐Z	Central America
NS	Playa Salamina, Nicaragua	R‐Z	Central America
PN	Playa Nancite, Costa Rica	P	–
PG	Playa Grande, Costa Rica	P	–
PO	Playa Ostional, Costa Rica	P	–
PMA	La Marinera, Panama	R‐Z	Central America

Note

Full sites include two‐letter country codes. Also indicated is whether site was originally included in the Rodríguez‐Zárate et al. (2018; R‐Z) data or present study (P), and population inferred by Rodríguez‐Zárate et al. (2018)

**FIGURE 1:**
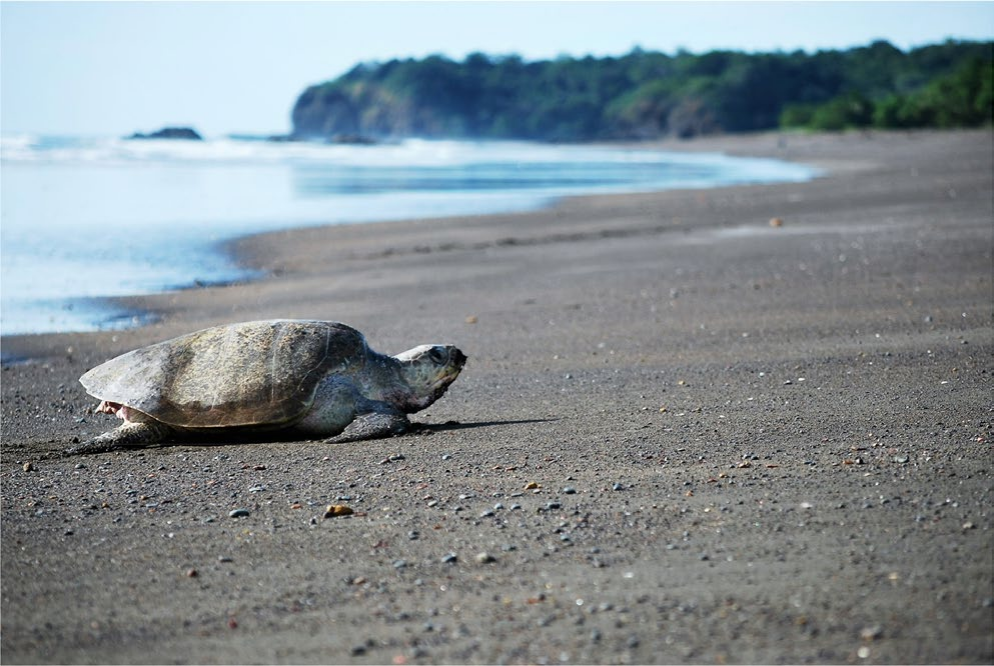
Nesting olive ridley (*Lepidochelys olivacea*) at Playa Ostional, Costa Rica. Although this female is alone on the beach, the site is known for arribada nesting. Photograph courtesy Quintin Bergman

MUs are not well defined for olive ridley sea turtles (*Lepidochelys olivacea*; Figure 1). Olive ridleys are the most abundant sea turtle globally (Abreu–Grobois & Plotkin^2008^), and entire ocean basins constitute the few existing MUs (see Komoroske et al., 2017 and references therein). Olive ridleys display unique reproductive traits relative to other sea turtle species (excluding the congeneric Kemp's ridley, *Lepidochelys kempii*) that influence their conservation status and population structure (Bernardo & Plotkin, 2007). Olive ridleys often nest *en masse* during “arribada” events, which may comprise tens of thousands of individual turtles (Bernardo & Plotkin, 2007). The size of arribada events lends support to olive ridleys' “threatened” (rather than endangered) status on the IUCN Red List (Abreu–Grobois & Plotkin 2008). Olive ridleys also likely display limited natal fidelity to nesting beaches relative to all other sea turtle species (Dornfeld, Robinson, Tomillo, & Paladino, 2015; Kalb, 1999). Sea turtles typically home to the region from which they hatched to reproduce, which engenders population structure around nesting beaches (Bowen & Karl, 2007). Mating offshore of arribada events may facilitate admixture if thousands of olive ridleys from distant beaches are involved (Jensen, Abreu‐grobois, Frydenberg, & Loeschcke, 2006). Both of these unique behaviors could weaken signatures of population structure that might be inferred from mtCR and microsatellite genotype data. However, adequate region‐wide genetic studies of olive ridleys have not been undertaken globally to assess population structure or determine MUs below the scale of entire ocean basins.

The Eastern Tropical Pacific olive ridley population is robust, with multiple arribada nesting sites and high‐density solitary nesting sites (López‐Castro & Rocha‐Olivares, 2005; Valverde et al., 2012). Despite their contemporary abundance, adult olive ridleys and eggs were extensively harvested in the Eastern Tropical Pacific in the mid–late 20th century (Márquez, Peñaflores, & Vasconcelos, 1996; Spotila, 2004) and adult olive ridleys constitute a large proportion of contemporary fisheries bycatch (Moore et al., 2009). The number of individuals participating in arribadas has also exhibited decades‐long declines (from millions to tens of thousands; Fonseca, Murillo, Guadamúz, Spínola, & Valverde, 2009). Costa Rica hosts two of the most prominent arribada beaches and index sites in the Eastern Tropical Pacific (Playa Nancite and Ostional) and is a global focal point for research on olive ridley biology and conservation. Playa Nancite historically hosted arribada assemblages as large as 115,000 individuals, which decreased into the early 21st century and has stabilized at ~8,000 individuals per arribada (Fonseca & Valverde, 2010). Playa Ostional has exhibited a recent increase in olive ridley abundance (Eguchi, Gerrodette, Pitman, Seminoff, & Dutton, 2007) and is estimated to have hosted assemblages as large as 476,550 individuals (Valverde et al., 2012). Since the late 1980s, locals have legally harvested ~20% of the eggs from arribada events, although local consumption was likely ongoing prior to this (Valverde et al., 2012).

There appears to be minimal population structuring within the Eastern Tropical Pacific Basin, and phylogeographic studies of olive ridleys have suggested that the Eastern Tropical Pacific population may be no more than 250,000–300,000 years old (Bowen et al., 1997; Jensen et al., 2013). Earlier genetic population assessments based on mtCR data indicated that olive ridleys nesting across the Baja California Peninsula (Mexico) may comprise a discrete MU (López‐Castro & Rocha‐Olivares, 2005). This finding was later supported by microsatellite analyses in a basin‐wide study that additionally proposed a weak but significant partition (*F*_ST_ = 0.028, *p* = .000) between Mexican and Central American populations across the entire Eastern Tropical Pacific (Rodríguez‐Zárate et al., 2018). Though comprehensive, the study did not survey genetic variability of key population index sites in Costa Rica (but did survey index sites in Mexico and Nicaragua, Table 1; Abreu‐Grobois & Plotkin, 2008; Bernardo & Plotkin, 2007). It is difficult to test for this Mexican–Central American split using mtCR haplotypes because haplotypes are not well reported for Central America, and the MUs (Mexican and Central American populations) proposed by Rodríguez‐Zárate et al. (2018) based on nuclear genetic variability are yet to be formally integrated into management frameworks. Further, while Rodríguez‐Zárate et al. (2018) found that environmental variables (namely oceanographic features such as the Costa Rica Dome) may play a role in this structuring, other factors such as relatedness and the effects of bottleneck events were not explored (although they were previously reported for the Mexican population; Rodríguez‐Zárate, Rocha‐Olivares, & Beheregaray, 2013).

We conducted this study to explore the nature and scale of population structuring in Costa Rican and Eastern Tropical Pacific olive ridleys using hierarchical and comparative population inference (i.e., clustering versus ordination population inference) to refine structuring beyond broad Mexican and Central American populations. We generated mtCR haplotype and nuclear microsatellite data from olive ridleys nesting at three sites in Costa Rica and inferred fine‐scale structuring, and then retrieved published microsatellite data from Rodríguez‐Zárate et al. (2018) to compare patterns in population structuring at different scales within the Eastern Tropical Pacific. To our knowledge, this is the first study since Bowen et al. (1997) to report mtCR haplotype data from olive ridleys south of Mexico. We compared mtCR haplotype data from Costa Rican olive ridleys to previously published data for Mexican olive ridleys to look for structuring across the Eastern Tropical Pacific. We subjected both microsatellite data sets to clustering and ordination population inference analyses, and investigated the mechanisms behind inferred structuring using metrics of population differentiation, bottleneck analysis, and relatedness analysis. This study furthers our understanding of Eastern Tropical Pacific olive ridley sea turtle biology, and provides valuable information to assist management conservation of the species in this region.

## 2 METHODS

### 2.1 Site and sampling description

**FIGURE 2:**
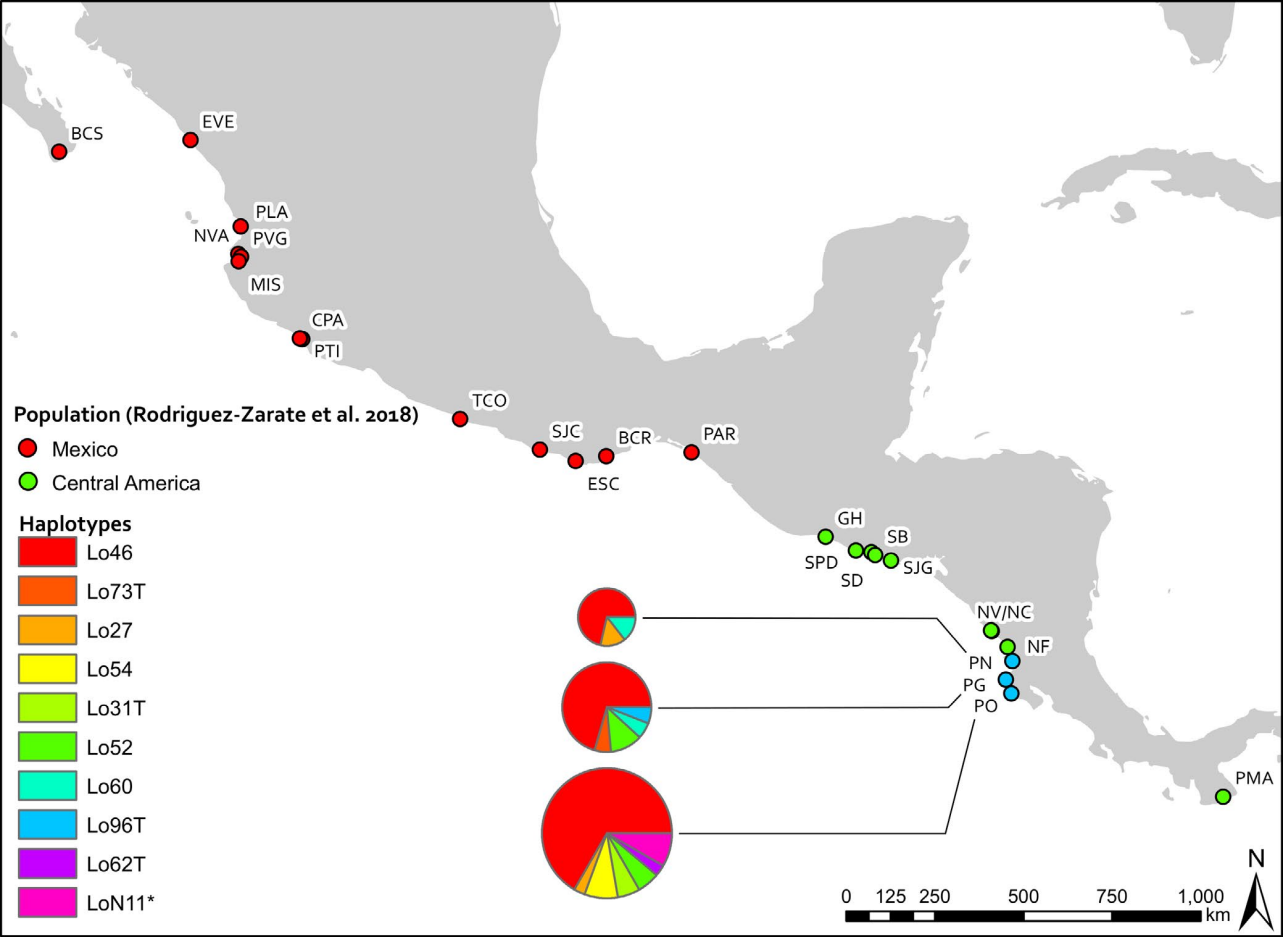
Map of Eastern Tropical Pacific and Costa Rican sampling sites and Costa Rican olive ridley mtCR haplotypes. Site abbreviations are explained in Table 1. Costa Rican haplotype frequencies for each site are represented by pie charts, and chart size corresponds to relative sample size. Haplotypes are named as per NMFS nomenclature (P. Dutton, *personal communication*). “*” indicates one haplotype that is in the NMFS nomenclature but could not be reliably named. “T” indicates haplotypes first reported in this study

Blood samples and skin samples from 118 olive ridley turtles collected in 1999 (Playa Nancite, *n* = 7; Figure 2; Clusella‐Trullas, Spotila, & Paladino, 2006), 2011–2012, and 2013–2014 (Playa Ostional, *n* = 78; Playa Grande, *n* = 33; Figure 2) were processed in 2014. All three sites host solitary nesting, but only Playa Grande does not host arribada nesting. Playa Grande is situated between Playas Nancite and Ostional and has been a national park since the early 1990s. Olive ridley nesting at Playas Nancite and Ostional is as described above. Playa Grande hosts approximately 70 olive ridley nests per year (350 nests and 285 individuals between 2009 and 2014; Dornfeld et al., 2015). Of the samples collected at Playa Ostional, 65 were arribada nesters and 13 were solitary nesters. All samples from Playa Nancite were from arribada nesters, and all samples from Playa Grande were from solitary nesters. We do not expect population allele frequencies to differ between sampling years due to the long generation times of sea turtles (~20–30 years; Spotila, 2004).

### 2.2 mtCR analysis

DNA was extracted from samples using Qiagen DNeasy Blood and Tissue Kits following the manufacturer's protocol and frozen at −20ºC. Samples were diluted to 25 ng/μl before PCR. Approximately 800 base pairs (bp) from the D‐loop of the mtCR were amplified for 60 turtles from Playas Nancite (*n* = 7), Ostional (*n* = 36; Arribada = 24, Solitary = 12), and Grande (*n* = 17) using primers LTEi9 (5’‐AGCGAATAATCAAAAGAGAAGG‐3’) and H950 (5’‐GTCTCGGATTTAGGGGTTTA‐3’; Abreu‐Grobois et al., 2006; Jensen et al., 2013). PCRs were conducted using Qiagen Taq PCR Master Mix Kits following the manufacturer's protocol. We used a thermocycling profile previously described by Jensen et al. (2013): denaturing at 94°C for 5 min, then 35 cycles of 45 s at 94°C, 45 s at 52°C, and 45 s at 72°C, followed by final extension at 72°C for 5 min. PCR products were run on agarose gels to ascertain quality and product size.

PCR products were purified using ExoSAP‐IT (Thermo) and sent to Genewiz (New Jersey, USA) for sequencing. Forward and reverse sequences were trimmed to approximately 800 bps, assembled, and aligned in Geneious v.11 (Kearse et al., 2012) using the CLUSTALW algorithm (Thompson, Gibson, & Higgins, 2003). Olive ridley mtCR haplotypes (~800bp) from prior studies (Bahri, Atmadipoera, & Madduppa, 2018; Bowen et al., 1997; Campista León et al., 2019; Jensen et al., 2013; López‐Castro & Rocha‐Olivares, 2005; Plot et al., 2012; Revuelta, 2015; Shanker, Ramadevi, Choudhury, Singh, & Aggarwal, 2004) were procured from GenBank and aligned with haplotypes from this study. New and existing haplotypes from Costa Rica were determined using DnaSP 6 (Rozas et al., 2017). All haplotypes were named as per National Marine Fisheries Services protocols (P. Dutton, *personal communication*). New haplotypes (*n* = 4) were confirmed by resequencing and deposited in GenBank (Accession nos. MK749418, MK749419, MK749420, and MK749421). Haplotype networks were generated from this study in TCS (Clement, Posada, & Crandall, 2000) and modified for publication using tcsBU (Múrias dos Santos, Cabezas, Tavares, Xavier, & Branco, 2016). Genetic diversity at all sites was quantified by calculating mean haplotype diversity (*H*) and mean nucleotide diversity (π) in Arlequin v.3.5 (Excoffier & Lischer, 2010).

### 2.3 Microsatellite analysis

We amplified eight microsatellite loci previously identified and characterized for olive ridleys (Aggarwal, Lalremruata, Velavan, Pavani Sowjanya, & Singh, 2008; Aggarwal et al., 2004) using fluorescently labeled (FAM and HEX) primers (Table S1). We forced the adenylation of PCR products by adding a linker sequence (CAGTCG‐) to the 5’ end of each reverse primer to facilitate genotyping. PCR cycling parameters followed the methods of Rodríguez‐Zárate et al. (2013) with annealing temperatures optimized for locus OR11. Touchdown PCR profiles consisted of 3 min at 94ºC followed by 35 cycles of 94ºC for 20 s; 61ºC‐53ºC until fifth cycle for 45 s; 72ºC for 1 min; and 10 min at 72ºC. Fragments were characterized using a 3730*xl* DNA Genetic Analyzer (Applied Biosystems) with a LIZ500 size standard at the DNA Analysis Facility at Yale University. Peaks were visualized using GeneMapper v.3.7 (Applied Biosystems) and scored manually by a single observer (J. Koval). Microsatellite genotypes are available in Dryad repository (https://doi.org/10.5061/dryad.c866t1g4f).

The number of alleles per locus (*k*), the number of effective alleles *Ne*, and observed and expected heterozygosity (*H_O_* and *H_E_*) were quantified using GenAlEx v.6.5 (Peakall & Smouse, 2006). Loci were tested for deviations from Hardy–Weinberg equilibrium and linkage disequilibrium using GENEPOP v.4.0.10 (Rousset, 2008; Rousset & Raymond, 1995). All samples were tested for heterozygote deficiency (evidence of null alleles) or heterozygote excess. A sequential Bonferroni correction was applied to account for multiple pairwise comparisons (Rice, 1989) in order to decrease the chance of type I error. A separate microsatellite data set for Eastern Tropical Pacific olive ridleys (10 loci amplified in 666 total individuals from 22 sites; Table 1; Rodríguez‐Zárate et al., 2018) was obtained from https://doi.org/10.5061/dryad.nj344m5, and descriptive statistics of those data may be found in Rodríguez‐Zárate et al. (2018). It should be noted that we did not combine data generated herein and those generated by Rodríguez‐Zárate et al. (2018), but conducted identical analyses with both data sets separately.

### 2.4 Population inference

Clustering (in STRUCTURE; Pritchard et al. 2000) and ordination (in *adegenet*; Jombart, 2008) population inference were used to assign individuals to genetically discrete populations (*K*) based on microsatellite genotypes. For Costa Rican olive ridleys, STRUCTURE was run 10 times for each *K* = 1–15, with a burn‐in of 50,000 generations and an MCMC of 100,000 generations. Each run assumed correlated allele frequencies (Falush, Stephens, & Pritchard, 2007) and historical admixture between putative populations (arribada and solitary nesting turtles from each site; Pritchard et al., 2000). Runs were repeated with and without sampling location as a prior (locprior). Rodríguez‐Zárate et al. (2018) ran STRUCTURE 20 times for each *K* = 1–22 (one *K* for each sampling site) with a burn‐in of 10,000 generations and an MCMC of 100,000. The authors also ran STRUCTURE assuming admixture between inferred populations, and with and without locprior. We analyzed both data sets in STRUCTURE with both sets of parameters (keeping *K* = 1–15 for Costa Rican olive ridleys and *K* = 1–22 for Eastern Tropical Pacific olive ridleys) to control for differences that might be attributed to different parameters, but runs of both parameters on each data set produced identical results within each data set. In addition to Rodríguez‐Zárate et al. (2018) full data set, we analyzed Mexican and Central American populations separately for internal structuring. STRUCTURE was run 20 times for each *K* = 1–22, with both parameter sets described above for Mexican and Central American olive ridleys.

STRUCTURE output files were analyzed in StructureSelector (Li & Liu, 2018) to determine the best estimate of *k* using multiple metrics. The estimated log probability of the data given a particular value of *K* (pr(X|Z,P), where X is the data and Z [*K*] is a grouping of individuals with P allele frequencies, allows the estimation of the most likely number of clusters (Pritchard et al., 2000). The ad hoc delta‐*K* method (Evanno, Regnaut, & Goudet, 2005) reports the second‐order rate of change of the log probability of each *K*, which typically peaks at the appropriate value of *K*. The admixture model calculates the fractional probability (*Q*) of individuals belonging to each population. Puechmaille’s (2016) four estimators (included in StructureSelector) base the likelihood of *K* on whether or not subpopulations (i.e., sampling sites) have at least 50% assignment to clusters for each *K*. Clusters that do not receive at least 50% assignment within subpopulations are defined as spurious, and lower the likelihood of that clustering configuration. These estimators are found to perform better than the log probability method in STRUCTURE (Pritchard et al., 2000) and the delta‐*K* method (Evanno et al., 2005).

A discriminant analysis of principal components (DAPC; Jombart et al., 2010) was run for both data sets using *adegenet* (Jombart, 2008) as implemented in R. DAPC was run with sampling sites as groups, and inferred clusters as groups. Genotype data were transformed into a coordinate format for principal component analysis (PCA) using read.genepop(). The most likely number of clusters was determined using find.clusters(), which employs k‐means clustering and a Bayesian information criterion to identify clusters. DAPC was run for each configuration (Costa Rican sites and inferred clusters, and Eastern Tropical Pacific sites and inferred clusters) using dapc(). As suggested by Jombart et al. (2010), 100% of the initial PCs were retained when identifying *K*, PCs accounting for ~ 80% of variance were retained during DAPC, and all axes of the DA were retained. Each DAPC was cross‐validated with optim.a.score() and rerun with suggested PCs and all axes to minimize error and overfitting (Jombart et al., 2010). For each configuration, the following optimal PCs were retained during DAPC: Costa Rican sampling sites, 14; Costa Rican inferred clusters, 4; Eastern Tropical Pacific sampling sites, 29; Eastern Tropical Pacific inferred clusters, 13.

### 2.5 Population differentiation and equilibrium

Pairwise *Φ*st (an *F*_ST_ estimator for mtCR data) and pairwise *θ*_ST_ (an *F*_ST_ estimator that accounts for uneven sampling; Weir & Cockerham, 1984) were quantified over 10,000 bootstrap replicates in Arlequin v. 3.5 (Excoffier & Lischer, 2010) for mtCR haplotypes from Costa Rican olive ridleys.

Costa Rican sampling sites and behaviors (solitary turtles from Playa Grande, solitary turtles from Playa Ostional, arribada turtles from Playa Ostional, and arribada turtles from Playa Nancite; i.e., Jensen et al., 2006), and Costa Rican and Eastern Tropical Pacific structuring inferred using clustering and ordination population inference were tested for population structure (*F*_ST_ and AMOVA; Pairwise *F*_ST_ and *D*) over 10,000 bootstrap replicates in Arlequin v. 3.5 (Excoffier & Lischer, 2010) and GenAlEx (Peakall & Smouse, 2006), respectively. *F*_IS_ was calculated to examine inbreeding for inferred structuring over 10,000 bootstrap replicates in Arlequin v. 3.5. Global Hardy–Weinberg equilibrium tests for heterozygote deficiency and excess were run for inferred structuring to determine whether it comprised randomly mating populations in GENEPOP v. 4.0.10 (10,000 step dememorization, 20 batches with 5,000 iterations; Rousset, 2008; Rousset & Raymond, 1995).

Rodríguez‐Zárate et al. (2018) report pairwise *F*_ST_ and *D* for Eastern Tropical Pacific sampling sites in their original publication. Alpha levels for pairwise *F*_ST_ and *D* were adjusted using a sequential Bonferroni correction for multiple testing.

### 2.6 Bottleneck analysis

A rapid decrease in population size leads to a reduction of the number of alleles present in the population, and therefore creates a heterozygosity deficiency (more heterozygotes are expected than actually exist). Rapid expansion after a bottleneck event leads to an increase in the number of alleles in the population, and therefore creates a heterozygosity excess (fewer heterozygotes are expected than actually exist). Recent extractive take from Eastern Tropical Pacific olive ridleys may have left such bottleneck signatures in genetic data from inferred populations, which should necessitate conservation actions. We therefore looked for bottleneck signatures in inferred structuring using BOTTLENECK (Cornuet & Luikart, 1996; Piry, Luikart, & Cornuet, 1999). BOTTLENECK can detect moderate bottlenecks with confidence for as many as 250 generations after they have occurred (Cornuet & Luikart, 1996) far longer than the approximately five to six generations of olive ridleys that have come into existence since the peak of take in the Eastern Tropical Pacific (Spotila, 2004). BOTTLENECK was run for 10,000 iterations of the two‐phase mutation model (TPM; Di Rienzo et al. 1994 as suggested for microsatellite data by Piry et al. (1999; 95% Stepwise Mutation Model [SMM; Ohta & Kimura, 2008] in the TPM with variance = 12). BOTTLENECK was also run with TPM settings suggested by Piry et al. in web documentation for the program (http://www1.montpellier.inra.fr/CBGP/software/Bottleneck/pub.html; 0% SMM in the TPM and variance = 36). The TPM is thought to be more representative of actual processes of mutation and evolution than other mutation models (Di Rienzo et al. 1994, Piry et al., 1999). The TPM is also more conservative than other models in inferring bottleneck events, as alleles that differ by more than one repeat still have a probability of coming from one mutational event, rather than multiple mutational events (Sainudiin, Durrett, Aquadro, & Nielsen, 2004). All BOTTLENECK runs were accompanied by tests for L‐shaped distributions of allele frequencies to determine whether any inferred structuring exhibited evidence of a bottleneck‐induced mode‐shift.

### 2.7 Relatedness analysis

Relatedness (*r*) is measured for two individuals on a 0 to 1 scale (0 being unrelated, 1 being identical) based on how much of the genome these two individuals are estimated to share (see Blouin, 2003, for a review of relatedness theory, methods, and studies). As sea turtles typically display natal breeding fidelity (with a margin of error but typically within MUs; Lohmann et al., 2008), we expect a higher probability of relatedness between individuals from within the same inferred cluster than between individuals from different inferred clusters. We therefore examined average pairwise relatedness within inferred structuring using two different algorithms (LRM: Lynch & Ritland, 1999; and QGM: Queller & Goodnight, 1989) over 10,000 iterations in GenAlEx (Peakall & Smouse, 2006). Queller and Goodnight's (QGM; 1989) estimator is a coefficient based only on the estimated identity by descent (IBD; Grafen, 1985). Lynch and Ritland (1999) estimator uses a regression calculation to determine relatedness coefficients for any pair of individuals based on shared IBD alleles, but can perform poorly if few related individuals are sampled, or if loci are too highly polymorphic (Blouin, 2003). Both estimators may also have high variances when few loci (*n* < 20) are used, but can provide a good estimation of relatedness between groups of individuals (Blouin, 2003; Queller & Goodnight, 1989). GenAlEx tests specifically for significantly high relatedness within groups (i.e., inferred structuring) relative to global relatedness, and we therefore focused on relative values of relatedness and the significance of inferred structuring relatedness rather than on specific thresholds of relatedness.

Further, we explored the potential for inferred structuring to comprise closely related individuals (parent–offspring pairs, full siblings, and half siblings) in COLONY v. 2.0.6.5 (Jones & Wang, 2010). First, to verify that both microsatellite data sets were powerful enough to exclude incorrect parent pairs (in light of the absence of known parents), we calculated P3Exc (the probability of excluding incorrect parent pairs when both parent genotypes are unknown) in GenAlEx for increasing combinations of loci for inferred structuring in Costa Rican and Eastern Tropical Pacific olive ridleys. We ran COLONY with all loci for each data set (*n* = 6 for Costa Rican data, *n* = 10 for Eastern Tropical Pacific data) for five “very long” runs of the full‐likelihood method of determining parentage and sibships with “very high” likelihood precision. We allowed for both male and female polygamy, as multiple paternity is commonly documented in sea turtles (see Lee, Schofield, Haughey, Mazaris, & Hays, (2018) for a review), and also allowed for inbreeding. We did not include a sibship prior. We examined the number of parent–offspring, full‐sibling, and half‐sibling pairs within and between inferred structuring groups to determine whether inferred structuring groups contained more parent–offspring or sibling pairs (i.e., family lineages) than pairs with members in two different groups.

## 3 RESULTS

### 3.1 Costa Rican mtDNA

We observed ten ~ 800 bp haplotypes from 60 turtles nesting at Playa Nancite (*n* = 7), Playa Grande (*n* = 17), and Playa Ostional (*n* = 36; Table 2; Figure 2 and Figure S1). Overall haplotype diversity (*H* = 0.5657 ± 0.0783 *SD*) and nucleotide diversity (π = 0.0014 ± 0.0012 *SD*) were comparable to those reported in other studies of Pacific olive ridleys (i.e., Campista León et al., 2019; Jensen et al., 2013; López‐Castro & Rocha‐Olivares, 2005) and did not vary between sites (as suggested by overlapping standard deviations; Table 2).

**TABLE 2:** Olive ridley mtCR haplotype frequencies from three nesting beaches in Costa Rica (PN: Playa Nancite; PG: Playa Grande; and PO: Playa Ostional) and overall (O) for this study

Site	*n*	*H* (±*SD*)	π (±*SD*)	Lo46	Lo73^T^	Lo27	Lo54	Lo31^T^	Lo52	Lo60	Lo96^T^	Lo62^T^	LoN11*
PN	7	0.5238 (±0.2086)	0.001128 (±0.001028)	5	0	1	0	0	0	1	0	0	0
PG	17	0.5074 (±0.1403)	0.001316 (±0.001042)	12	1	0	0	0	2	1	1	0	0
PO	36	0.6151 (±0.954)	0.001583 (±0.001155)	24	0	1	3	2	2	0	0	1	3
O	60	0.5657 (±0.0783)	0.001434 (±0.01061)	41	1	2	3	2	4	2	1	1	3

Note

Also shown are the number of individuals from each site (*n*), mean (±*SD*) haplotype diversity (*H*), and mean (±*SD*) nucleotide diversity (π). “T” indicates haplotypes first reported at nesting beaches from this study. “*” indicates one haplotype that is known to NMFS but could not be named with certainty.

There was no evidence that any of the three sites were genetically distinct from each other as determined by pairwise *Φ*_ST_ (*Φ*_ST_ = −0.05–0.00021, *p* = .37–0.79), pairwise *θ*_ST_ (*θ*_ST_ = −0.03–0.02, *p* = .61–0.79), and AMOVA (variance explained = −0.21%, *F*_ST_=−0.02, *p* = .65). We compared our data to those published for Mexican olive ridleys (Playa de Ceuta, Sinaloa; Campista León et al., 2019). Pairwise *Φ*st (*Φ*_ST_ = −0.017–0.00571, *p* = .32–.81), pairwise *θ*_ST_ (*θ*_ST_ = −0.02–0.0043, *p* = .41–0.78), and AMOVA (variance explained = −0.13%, *F*_ST_ = 0.001, *p* = .57) did not suggest divergence between any sampling sites. We were unable to compare our data to other published data for Mexican olive ridleys (i.e., López‐Castro & Rocha‐Olivares, 2005) due to the lack of a consistent, systematic nomenclature for olive ridley haplotypes (P. Dutton, *personal communication*). However, haplotype Lo46 comprised 68% of the haplotypes we found, which is consistent with (albeit lower than) López‐Castro and Rocha‐Olivares (2005) findings from Mexican olive ridleys (~90%) and suggests a lack of mitochondrial differentiation between Mexican and Costa Rican nesting assemblages.

### 3.2 Costa Rican microsatellite analysis

All eight loci amplified successfully, albeit not in every individual (97.1%±3 *SD* amplification success; Table S1, also see supplementary data). We did not detect any deviations from HWE, but three loci (OR7, OR16, and OR22) were found to be in linkage disequilibrium (*p* < .000001). OR7 and OR22 were excluded from all analyses, bringing the effective number of loci down from eight to six. The remaining six loci still comprised a relatively powerful and allele‐rich (*k*) data set: OR2 (*k* = 12), OR4 (*k* = 19), OR9 (*k* = 7), OR11 (*k* = 19), OR16 (*k* = 12), and OR18 (*k* = 6). Pairwise *F*_ST_ (F_ST_ = 0.005–0.009, *p* = .58–0.90) and *D* (*D* = −0.025–0.020, *p* = .55–0.91) did not suggest divergence between solitary nesters at Playa Ostional, as compared to arribada nesters at Playa Ostional, as well as to the solitary nesters at Playa Grande. Turtles from Playa Nancite were significantly diverged from all other Costa Rican groups (*F*_ST_ = 0.05–0.061, *p* = .0–.002; *D* = 0.118–0.145, *p* = .00–.013), but this may have been due to six out of seven of these turtles missing data at some loci. AMOVA indicated no differentiation among Costa Rican sampling sites or behaviors (variance explained = −0.32%, *F*_ST_ = −0.003, *p* = 1.00).

### 3.3 Population inference

In Costa Rican olive ridleys, analysis of clustering population inference results initially suggested *K* = 2 as the most likely population structure, but assignment plots for *K* = 2 suggested admixture. We therefore interpreted *K* = 1 as the most likely structuring scheme. Rodríguez‐Zárate et al. (2018) reported two clusters representing Mexican and Central American populations for Eastern Tropical Pacific olive ridleys as inferred using STRUCTURE. When analyzing Mexican and Central American populations separately, we found weak structure within Mexican olive ridleys with the location prior model enabled that discriminated turtles nesting at Puerto Arista from all other turtles (PAR; Figure S2). AMOVA and pairwise *F*_ST_ confirmed significant, moderate structuring between PAR and other Mexican sites (AMOVA: among population variance explained = 6.7%, *F*_ST_ = 0.067, *p* < .001; pairwise *F*_ST_: *F*_ST_ = 0.066, *p* < .001). Similar hierarchical analysis using STRUCTURE in Mexico without locprior, as well as in Central America with and without locprior, did not allow us to discern substructuring.

**FIGURE 3:**
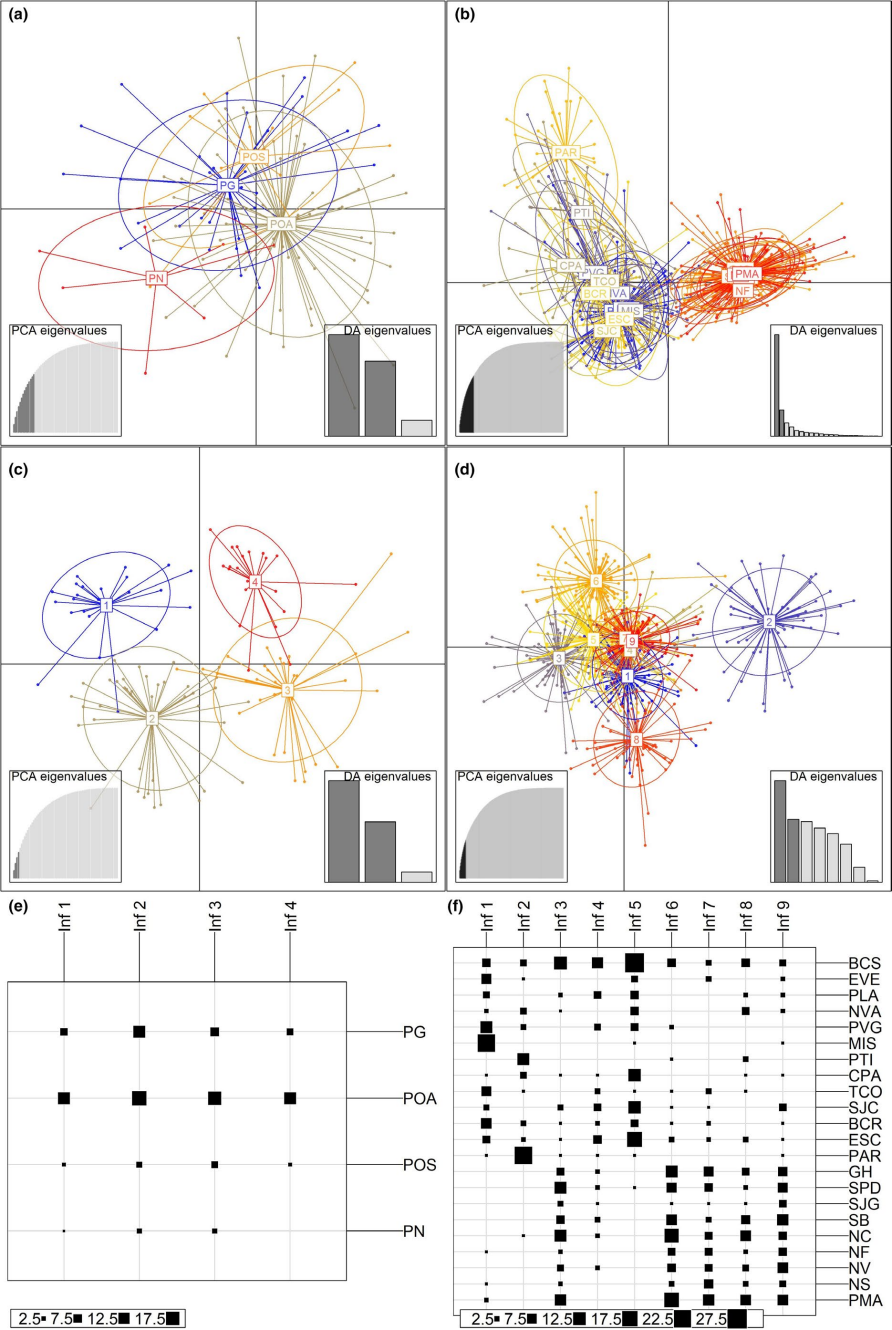
DAPC (ordination) scatter plots and box plots from Costa Rican (a, c, e) and Eastern Tropical Pacific (b, d, f) olive ridleys analyzed by nesting sites (a, b) and by inferred clusters (c, d). Scatter plot inertia ellipses summarize dispersion from the centroids of nesting sites and inferred clusters, which are labeled by nesting site or cluster number. Box plots show the number of individuals (represented by box sizes, bottom left of each plot) from each site (along right y‐axes) that are assigned to each inferred cluster (“Inf #”; along top x‐axes). In a and e: PG is Playa Grande, POA is Playa Ostional Arribada, POS is Playa Ostional Solitary, and PN is Playa Nancite. In b and f, abbreviations follow Rodríguez‐Zárate et al., 2018; Table 1). In F, sites are in North to South order. Note that PAR is the southernmost site in the Mexican population and GH the northernmost site in the Central American population

Ordination population inference by sampling site for Costa Rican olive ridleys produced similar results to clustering population inference. Assignment proportions to sites and behaviors were low (mean = 0.59 ± 0.04 *SE*), and turtles grouped by site and behavior failed to discriminate from one another (Figure 3a). However, ordination population inference by inferred clusters elucidated four discrete clusters with high assignment proportions (mean = 0.97 ± 0.02 *SE*), which contained individuals from all sites and behaviors (Figure 3c, e). Clusters were significantly diverged from one another (pairwise *F*_ST_ = 0.069–0.095, pairwise *D* = 0.239–0.315, *p* < .001; Table 3a) and effectively partitioned genetic variation in Costa Rica (AMOVA: variance explained = 8.3%, *F*_ST_ = 0.08, *p* < .001). All clusters displayed negligible *F*_IS_ (*F*_IS_ < 0; Table 4), but clusters 2, 3, and 4 had significant heterozygote excess in global HWE exact tests (*p* < .0001, *p* = .0045, and *p* = .0229, respectively; Table 4).

**TABLE 3:** Pairwise population differentiation indices (*F*_ST_, Wright 1951; Jost, 2008) for Costa Rican (a) and Eastern Tropical Pacific (b) ordination clusters calculated after 10,000 permutations

(a)	1	2	3	4
1		0.081	0.080	0.093
2	0.259		0.095	0.070
3	0.239	0.310		0.074
4	0.315	0.292	0.265	

(b)	1	2	3	4	5	6	7	8	9
1		0.067	0.054	0.049	0.052	0.065	0.050	0.058	0.065
2	0.303		0.082	0.056	0.062	0.074	0.061	0.066	0.073
3	0.191	0.310		0.091	0.059	0.084	0.029	0.074	0.089
4	0.318	0.373	0.507		0.037	0.037	0.040	0.041	0.039
5	0.241	0.300	0.231	0.250		0.054	0.049	0.055	0.061
6	0.359	0.422	0.388	0.301	0.318		0.044	0.051	0.052
7	0.252	0.319	0.111	0.309	0.271	0.280		0.041	0.045
8	0.290	0.344	0.311	0.309	0.298	0.324	0.237		0.053
9	0.323	0.377	0.376	0.283	0.328	0.324	0.257	0.303	

Note

Pairwise *F*_ST_ values are above the diagonal (top right), and pairwise *D* values are below the diagonal (bottom left). All values were significant (alpha = 0.0125 and 0.00139, respectively).

**TABLE 4:** Bottleneck results (TPM, sign test and Wilcoxon test), *F*_IS_ values, and Hardy–Weinberg equilibrium heterozygote excess (*E*), and deficiency (*D*) *p*‐values, for Costa Rican and Eastern Tropical Pacific (Mexico, Central America) olive ridleys and ordination clusters

Site	TPM Sign 0	TPM Wilcoxon 0	TPM Sign 95	TPM Wilcoxon 95	*F*_IS_	*E*	*D*
Costa Rica	0.316	**0.039 (E)**	0.061	**0.019**	–	–	–
Cluster 1	0.168	0.641	0.054	**0.019**	−0.307	0.1671	0.8329
Cluster 2	0.585	0.743	0.054	0.055	−0.296	**0.0000**	1.0000
Cluster 3	0.578	1.000	0.056	**0.027**	−0.221	**0.0045**	0.9955
Cluster 4	0.599	0.382	**0.001**	**0.004**	−0.192	**0.0229**	0.9771
Mexico	0.374	0.921	**0.000**	**0.000**	–	–	–
Central America	0.622	0.492	**0.016**	**0.014**	–	–	–
Cluster 1 (M)	0.369	0.275	0.061	**0.024**	**0.099**	1.0000	**0.0000**
Cluster 2 (M)	0.168	0.3227	**0.002**	**0.003**	**0.094**	1.0000	**0.0000**
Cluster 4 (M)	**0.047 (E)**	**0.0419 (E)**	0.599	0.846	**0.107**	1.0000	**0.0000**
Cluster 5 (M)	0.166	0.845	0.065	**0.024**	**0.109**	1.0000	**0.0000**
Cluster 3 (C)	0.607	0.556	0.065	**0.014**	**0.075**	1.0000	**0.0000**
Cluster 6 (C)	0.617	0.556	0.396	0.432	0.059	1.0000	**0.0000**
Cluster 7 (C)	0.352	0.375	0.062	**0.014**	0.019	1.0000	**0.0000**
Cluster 8 (C)	0.354	0.769	0.063	**0.014**	−0.012	1.0000	**0.0000**
Cluster 9 (C)	0.365	0.492	0.065	0.130	−0.059	1.0000	**0.0000**

Note

Bottleneck measures were calculated over 10,000 bootstrap replicates. In bottleneck headings, “0” indicates 0% SMM in the TPM, “95” indicates 95% SMM in the TPM. Bold typeface indicates significant values (alpha = 0.05). (*E*) indicates significant heterozygote excess detected by BOTTLENECK. Eastern Tropical Pacific ordination clusters are ordered by Mexican (M) and Central American (C) populations.

Ordination population inference by nesting site for Eastern Tropical Pacific olive ridleys confirmed clustering population inference results: Mexican and Central American nesting beaches split along the first axis, PAR separated from other Mexican nesting beaches, and the Central American nesting beaches displayed admixture (Figure 3b). However, assignment proportions were low (mean = 65.3 ± 0.002 *SE*).

Ordination population inference elucidated 9 discrete clusters with high assignment proportions (mean = 0.99 ± 0.003 *SE*; Figure 3d) in Eastern Tropical Pacific olive ridleys. Clusters largely aligned with Mexican and Central American populations, but contained individuals from multiple nesting sites, some as distant as ~ 3,500 km along the coast (i.e., Baja California del Sur [BCS] and Panama [PMA]), within both Mexican and Central American populations (Figure 3f). AMOVA showed that ordination population inference clusters were moderately differentiated from each other (variance explained = 10.37%, *F*_ST_ = 0.103, *p* < .001) and pairwise *F*_ST_ (0.037–0.091) and *D* (0.111–0.507) confirmed that all clusters were significantly different from one another (*p* < .001 in all cases; Table 3b). Primarily Mexican clusters 1, 2, 4, and 5, and primarily Central American cluster 3 displayed elevated *F*_IS_ (*F*_IS_ = 0.075–0.109, *p* < .0001; Table 4). All clusters displayed significant heterozygote deficiencies in global HWE exact tests (*p* < .0001; Table 4).

### 3.4 Bottleneck analysis

BOTTLENECK results varied depending on the proportion of SMM in the TPM, and on the test used to validate the significance of results. In general, TPM with 95% SMM inferred more population expansion after bottleneck events than TPM with 0% SMM, which only inferred one instance of heterozygosity excess (Table 4). The two‐tailed Wilcoxon test conferred significance (alpha = 0.05) on heterozygosity deficiencies slightly more often than the sign test, specifically in Central American ordination population inference clusters from Eastern Tropical Pacific olive ridleys. However, both tests largely agreed on bottleneck significance, or the lack thereof.

With no SMM in the TPM, there were no inferred bottleneck events in either data set. This may be due to the constraints and limitations of the mutation models used in BOTTLENECK (Luikart, Allendorf, Cornuet, & Sherwin, 1998, Piry et al., 1999; Putman & Carbone, 2014). With 95% SMM in the TPM, there was still no evidence of bottleneck events in Costa Rican olive ridleys (Table 4). However, BOTTLENECK found that both Mexican and Central American populations had significant heterozygote deficiency (*p* = .00098 and .014 respectively; Table 4). The Wilcoxon test detected bottlenecks in three out of four Mexican ordination population inference clusters, and three out of five Central American ordination population inference clusters (*p* < .05; Table 4). However, the sign test only detected a bottleneck in cluster #2 (*p* < .05; Table 4). Despite this, none of the L‐shaped distribution tests suggested mode‐shifts in allele frequencies.

### 3.5 Relatedness analysis

LRM and QGM showed agreement in general patterns of relatedness, but differed in exact values of relatedness within nesting sites and putative populations. In general, LRM was more conservative than QGM. In Costa Rica, relatedness was negligible overall (Table 5). Relatedness was significantly high (*p* < .001) within ordination population inference clusters, and ranged from 0.310 to 0.570 (LRM) and 0.053 to 0.235 (QGM). Relatedness was higher in Mexican and Central American populations overall than in Costa Rica (Table 5). Relatedness within Eastern Tropical Pacific ordination population inference clusters was always higher than global relatedness (*p* < .001, LRM: 0.018–0.054, QGM: 0.095–0.311) and comparable to Mexican and Central American populations in all clusters, save for cluster 4 (QGM = 0.001).

**TABLE 5:** Relatedness measures (LRM, Lynch & Ritland, 1999; QGM, Queller & Goodnight, 1989) and full‐sibling (FS) and half‐sibling (HS) counts for Costa Rican and Eastern Tropical Pacific (Mexico, Central America) olive ridleys and ordination clusters. Relatedness measures were calculated over 10,000 bootstrap replicates

Site	LRM	QGM	FS		HS	
Costa Rica	−0.008	−0.006	–	–	–	–
Cluster 1	**0.057**	**0.235**	3/4	(0.75)	3/4	(0.75)
Cluster 2	**0.036**	**0.145**	7/14	(0.5)	7/14	(0.5)
Cluster 3	**0.031**	**0.225**	10/12	(0.83)	10/12	(0.83)
Cluster 4	**0.056**	**0.053**	3/6	(0.5)	3/6	(0.5)
Mexico	**0.013**	**0.022**	–	–	–	–
Central America	**0.024**	**0.044**	–	–	–	–
Cluster 1 (M)	**0.026**	**0.194**	6/5	(1.2)	6/5	(1.2)
Cluster 2 (M)	**0.054**	**0.213**	6/3	(2)	6/3	(2)
Cluster 4 (M)	**0.018**	0.001	10/6	(1.67)	10/6	(1.67)
Cluster 5 (M)	**0.025**	**0.154**	6/6	(1)	6/6	(1)
Cluster 3 (C)	**0.030**	**0.311**	4/8	(0.5)	4/8	(0.5)
Cluster 6 (C)	**0.044**	**0.117**	11/6	(1.83)	11/6	(1.83)
Cluster 7 (C)	**0.019**	**0.095**	8/6	(1.33)	8/6	(1.33)
Cluster 8 (C)	**0.027**	**0.134**	4/1	(4)	4/1	(4)
Cluster 9 (C)	**0.035**	**0.166**	4/1	(4)	4/1	(4)

Note

“/” in sibling counts denotes sibship counts within each cluster on the left, and between that cluster and other clusters on the right. Parenthetical values are the fractions of these internal versus external sibships. Bold typeface indicates significant values (alpha = 0.05, *p* < .001). Eastern Tropical Pacific ordination clusters are ordered by Mexican (M) and Central American (C) populations.

Complete exclusion of incorrect parent pairs (P3Exc = 1.00) was found when including four loci in Costa Rican ordination population inference clusters and five loci in Eastern Tropical Pacific ordination population inference clusters, which suggests that both data sets were appropriate for analysis in COLONY with all loci included. COLONY results for individual ordination population inference clusters are found in Table 5. COLONY did not identify parent–offspring relationships in either data set. In Costa Rican olive ridleys (*n* = 118), COLONY identified 41 full‐sibling pairs (23 pairs within ordination population inference clusters and 18 pairs between ordination population inference clusters) and 399 half‐sibling pairs (137 pairs within ordination population inference clusters and 262 pairs between ordination population inference clusters). In Eastern Tropical Pacific olive ridleys (*n* = 666), COLONY identified 81 full‐sibling pairs (59 pairs within ordination population inference clusters and 21 pairs between ordination population inference clusters) and 1,278 half‐sibling pairs (524 pairs within ordination population inference clusters and 754 pairs between ordination population inference clusters).

## 4 DISCUSSION

Olive ridleys were thought to display minimal population structuring within ocean basins (Bowen et al., 1997; Bowen & Karl, 2007) due in part to their low nesting site fidelity (Kalb, 1999) and broad foraging ranges (Plotkin, 2010). While we show that mtCR haplotypes are unstructured both locally and regionally in Eastern Tropical Pacific olive ridleys, ordination of nuclear microsatellite data suggests moderate, cryptic genetic structuring composed of related individuals within and between previously identified Mexican and Central American subpopulations (Rodríguez‐Zárate et al., 2018). Our results corroborate previous work showing basin‐wide connectivity and shallow population structure in Eastern Tropical Pacific olive ridleys (Bowen et al. 1997, Jensen et al., 2013; Rodríguez‐Zárate et al., 2018), but additionally suggest that select family lineages appear to have proliferated and dispersed throughout this region. We discuss these results and their implications for olive ridley and general sea turtle biology, particularly in light of historical anthropogenic take and mass synchronous arribada nesting.

### 4.1 Limitations

The analyses and results presented here were not without limitations. We were unable to directly compare the microsatellite data generated here and those generated by Rodríguez‐Zárate et al. (2018). This would require costly and time‐intensive calibrations, and we believe our side‐by‐side analyses are still of value toward understanding olive ridley population structure. We used relatively few microsatellite loci (*n* = 6) to study population structure among Costa Rican olive ridleys. Six loci have previously been used to study olive ridley population structure (Jensen et al., 2006), but Jensen et al. (2006) did not examine relatedness or population bottlenecks. Relatedness estimates improve in accuracy with increasing loci (Blouin, 2003), and BOTTLENECK may require more than six loci to adequately identify population bottlenecks (Peery et al., 2012; Williamson‐Natesan, 2005). Further, bottleneck signatures may be confounded by relatedness among individuals, which in some cases may lead to similar heterozygote deficiencies (as seen in some ordination population inference clusters here; Table 4). We therefore cautiously interpret relatedness and BOTTLENECK results from Costa Rican olive ridleys.

### 4.2 Connectivity

Analyses of mtCR haplotypes did not support or refine population structure in Costa Rican or Eastern Tropical Pacific olive ridleys. This may be due in part to relatively recent colonization of the Eastern Tropical Pacific by olive ridleys. Chelonian mitochondrial DNA has been shown to accrue mutations on a scale of tens of thousands to hundreds of thousands of years (Avise, Bowen, Lamb, Meylan, & Bermingham, 1992). Past phylogeographic studies of olive ridleys have suggested that the Eastern Tropical Pacific population may be no more than 250,000–300,000 years old (Bowen et al., 1997; Jensen et al., 2013). Thus, while at least 14 mtCR haplotypes are reliably documented from Eastern Tropical Pacific olive ridleys (Bowen et al., 1997; Jensen et al., 2013; López‐Castro & Rocha‐Olivares, 2005, Campista León et al., 2019), mutations accrue slowly and it is likely that no haplotypes have established themselves at a level similar to the basal haplotype (Lo46). We were unable to compare our mtCR haplotype data to all studies reporting haplotypes from Eastern Tropical Pacific olive ridleys (i.e., López‐Castro & Rocha‐Olivares, 2005) due to inconsistencies and omissions in naming and reporting of mtCR haplotypes for olive ridleys globally. Researchers and managers are increasingly focusing on olive ridley population structure, and such studies will benefit from a consensus, global nomenclature for olive ridley mtCR haplotypes. It has been 20+ years since the last global phylogeography of ridley turtles (genus *Lepidochelys*) was published (Bowen et al., 1997; but see Hahn, 2013), and a new effort to study ridley phylogeography will necessitate better organization of mtCR haplotypes.

Failure of mtCR haplotypes to refine population structure is also likely due to broad connectivity in Eastern Tropical Pacific olive ridleys. Previous studies have suggested that Eastern Tropical Pacific olive ridleys display little population structuring (Bowen et al., 1997; Jensen et al., 2013; Rodríguez‐Zárate et al., 2018) and site fidelity (Dornfeld et al., 2015; Kalb, 1999), and studies have found only weak structuring at broad scales (i.e., *F*_ST_ = 0.02, Rodríguez‐Zárate et al., 2018). Our analysis of microsatellite data also suggests broad connectivity across the Eastern Tropical Pacific, as we were unable to refine population structure below Mexican and Central American populations suggested by Rodríguez‐Zárate et al., 2018; save for at PAR; Figure S2), ordination by nesting sites failed to discriminate individuals with high reassignment proportions, ordination population inference clusters (discussed in‐depth below) contained individuals from distant beaches, and sibship analyses identified full and half siblings from sites as distant as BCS and PMA, nearly the entire range of olive ridley nesting in the Eastern Tropical Pacific. Individual females that nest with limited site fidelity may produce offspring that contribute to genetic homogeneity in the region, especially if those individuals then disperse vast distances from their natal beaches when reproducing. Further, males are not tied to nesting beaches and are known to be nomadic when mating in other sea turtle species (i.e., Roberts, Schwartz, & Karl, 2004, Carreras et al., 2007). Male‐mediated gene flow likely contributes to observed connectivity in Eastern Tropical Pacific olive ridleys. Finally, arribada events likely facilitate connectivity in Eastern Tropical Pacific olive ridleys (Jensen et al., 2006). There were historically six arribada beaches in the Eastern Tropical Pacific (Montero, Rincon, Heppell, & Hall, 2016), although some assemblages were extirpated in the 20th century due to extensive anthropogenic take of turtles (Spotila, 2004). Arribada beaches were and are foci for hundreds of thousands of breeding olive ridleys, and likely have fostered and continue to foster broad genetic connectivity in Eastern Tropical Pacific olive ridleys.

### 4.3 Lineages

Despite broad connectivity and weak large‐scale structuring, ordination population inference produced cryptic clusters in both Costa Rican and Eastern Tropical Pacific microsatellite data. These clusters presented with moderate *F*_ST_ and *D* (Table 3A and B), but also had notable internal relatedness (Table 5). Upon further examination, all of the Eastern Tropical Pacific clusters had significant heterozygote deficiency, five of the Eastern Tropical Pacific clusters had positive *F*_IS_, and all Costa Rican and Eastern Tropical Pacific clusters contain full‐ and half‐sibling pairs (Tables 4 and 5). Eastern Tropical Pacific clusters in particular contain more full‐sibling pairs within than between clusters, save for cluster 3 (Table 5). These results suggest that ordination population inference did not identify subpopulations, but instead identified family lineages in Eastern Tropical Pacific olive ridleys. Members of these family lineages primarily correspond to Mexican and Central American subpopulations, but these lineages also corroborate broad connectivity in Eastern Tropical Pacific olive ridleys as evidenced by their basin‐wide geographic distribution, and the ubiquitous presence of full‐ and half‐sibling pairs between clusters.

In Mexico, these lineages persisted despite intensive take, which may have engendered genetic bottleneck signatures (Márquez et al., 1996; Spotila, 2004; Table 4). Reduced genetic diversity due to bottlenecking may have accentuated differential survival and genetic signatures of family lineages in Mexico, hence positive *F*_IS_ and heterozygote deficiency (Table 4), and relatively high proportions of intra‐ versus intercluster full‐ and half‐sibling pairs in all primarily Mexican ordination population inference clusters (Table 5). These clusters even exhibited some spatial heterogeneity (Figure 3f), which may only be apparent due to reduced genetic diversity in Mexican olive ridleys overall, and further highlight relatively reduced connectivity between Mexican lineages and other lineages.

In Central America, take of eggs and adult turtles was not as severe as in Mexico (Spotila, 2004). While significant heterozygote deficiency and internal sibship pairs (particularly full siblings; Table 5) still suggest primarily Central American ordination population inference clusters represent family lineages, these lineages do not exhibit the degree of positive F_IS_ as do all Mexican lineages (save for cluster 3; Table 4). Central American lineages also display consistently lower proportions of intra‐ versus intercluster half‐sibling pairs (Table 5). The spatial homogeneity of Central American ordination population inference clusters (Figure 3f), negligible *F*_IS,_ and many intercluster half‐sibling pairs suggests relatively high genetic connectivity between Central American lineages and other lineages. This connectivity, as mentioned above, may be facilitated by the presence of four arribada sites in Central America, three of which (Playa La Flor, Nicaragua; Playas Nancite and Ostional, Costa Rica) are in close proximity (Montero et al., 2016).

Costa Rican ordination population inference clusters display similar characteristics to Central American ordination population inference clusters. Relatively high relatedness and the presence of intracluster sibships suggest these clusters also comprise family lineages. However, negligible *F*_IS_, heterozygote excess, and low proportions of intra‐ versus intercluster sibships suggest substantial connectivity between Costa Rican lineages. There is perhaps more connectivity demonstrated by Costa Rican lineages than by those in Central American lineages. This may be due to limited anthropogenic take of turtles in Costa Rica, and to the density of nesting beaches, and arribada beaches in particular, in Costa Rica (Montero et al., 2016; Spotila, 2004). The two arribada sites sampled in the present study are located within 100 km of each other (Figure 2) and may have a regional draw for breeding olive ridleys, who then mate with individuals from Mexican and Central American populations and other family lineages. However, this enhanced connectivity may be an artifact of using fewer loci (*n* = 6 in Costa Rica, *n* = 10 in Mexico and Central America). Further, there is no reason to suspect that Costa Rican olive ridleys would not be part of broader, Central American lineages, and it is possible that Costa Rican lineages would display the same connectivity as Central American lineages if microsatellite genotypes were comparable between studies.

### 4.4 Implications

The existence of broadly distributed family lineages is a first and previously unreported manifestation of vast dispersal and limited site fidelity in Eastern Tropical Pacific olive ridleys. The spatial distances between sampled nesting siblings and half siblings reported here may represent consistently large nesting remigrations by Eastern Tropical Pacific olive ridleys (i.e., inferred mothers from sibships). In one singular case (maternal half siblings sampled in BCS and PMA), the inferred mother may have completed one of the largest known remigrations by a nesting female sea turtle (~3,500 km by sea). It is equally probable that at least one of the half siblings from this pair nested quite far from its natal beach when it was sampled by Rodríguez‐Zárate et al. (2018).

There may be evolutionary precedent for the broad dispersal and distribution of Eastern Tropical Pacific olive ridley lineages. Spreading out nests over space and time mitigates the impacts of disturbances such as tropical storms and predation on individual fitness (Dewald and Pike 2014). Broadly distributed siblings may be product of nesting females distributing clutches to mitigate losses to their individual reproductive success. Dispersal away from natal beaches to nest reduces competition between parents and offspring, and in this case, siblings (Ronce, Gandon, & Rousset, 2000). Eastern Tropical Pacific olive ridleys are less likely to compete for nesting sites with kin if they disperse vast distances from natal beaches to reproduce. Dispersal also reduces the probability of inbreeding, which may ultimately reduce individual fitness (Perrin & Goudet, 2001) and may ensure that lineages persist despite stochastic, but spatially isolated, disturbances. For instance, olive ridleys from a primarily Central American lineage that were sampled while nesting in Mexico may survive disturbances that impact Central America, and continue contributing offspring to that lineage undisturbed. There may be some individual olive ridleys who never display natal fidelity as a consequence of this pressure to disperse from kin. These individuals would constitute fascinating exceptions to the natal‐homing paradigm known for sea turtles (Lohmann et al., 2008), if genetic capture–mark–recapture (i.e., Dutton & Stewart, 2013) or neonate telemetry (i.e., Mansfield, Wyneken, Porter, & Luo, 2014) could be implemented in the Eastern Tropical Pacific to identify nonhoming olive ridleys. Such studies may even provide novel insight into the physiological or molecular mechanisms behind natal homing. Finally, dispersal may also be a function of relatively large population sizes in Eastern Tropical Pacific olive ridleys (Fonseca & Valverde, 2010; Valverde et al., 2012), and individuals may have historically benefitted from dispersing away from more crowded beaches to preserve individual fitness (i.e., Honarvar, Spotila, & O’Connor, 2011; but see Bernardo & Plotkin, 2007 for a discussion of the evolution of arribada nesting). If this is the case, lineage dispersal should vary in other olive ridley and sea turtle populations according to their population size, a phenomenon with management implications that is worth investigating.

Genetic population and relatedness studies provide important insight into contemporary population structure and connectivity in wildlife species (Reid, Thiel, Palsbøll, & Peery, 2016; Schunter, Pascual, Garza, Raventos, & Macpherson, 2014) that if incorporated into management plans can help to reduce the impact of widespread threats and support the development of targeted conservation actions. We provide evidence of broad genetic connectivity and the existence of family lineages in Eastern Tropical Pacific olive ridleys, which explicitly connect distant nesting sites this region. This is of importance for management and conservation of Eastern Tropical Pacific olive ridleys given increasing pressure by unregulated fisheries and other threats in this region (Dapp, Arauz, Spotila, & O'Connor, 2013; Hope, 2002; Moore et al., 2009). Further knowledge of migratory routes used by olive ridleys, gained via satellite telemetry, will also be necessary to better understand how to protect olive ridleys at sea and to maintain genetic diversity, population structure, and family lineages in this region over time. Eastern Tropical Pacific olive ridley lineages merit investigation, and studies that examine individual fitness in light of kin dispersal in multiple lineages over multiple generations would provide insight into the evolutionary drivers behind lineage persistence and dispersal.

As mentioned before, genetic capture–mark–recapture, and neonate and adult satellite telemetry of males and females, throughout entire nesting seasons, will aid in elucidating the extent of natal homing and breeding fidelity in Eastern Tropical Pacific olive ridleys. It is as of yet unclear to what extent female olive ridleys display limited site fidelity between nesting events (perhaps traveling thousands of kilometers), or whether to some extent olive ridleys (including breeding males) do not exhibit natal homing for reproduction. Family lineages may underlie known population structure in other sea turtle species. Future studies that integrate molecular, spatial, and behavioral ecology techniques to investigate this phenomenon will provide novel insight for management of populations in these highly mobile species and will have an impact on our current understanding of sea turtle behavioral and spatial ecology.

## CONFLICT OF INTEREST

None declared.

## AUTHOR CONTRIBUTION

**Ian Silver‐Gorges:** Formal analysis (lead); Methodology (lead); Software (lead); Validation (lead); Visualization (lead); Writing‐original draft (lead); Writing‐review & editing (lead). **Julianne Koval:** Data curation (equal); Formal analysis (supporting); Methodology (supporting); Writing‐review & editing (supporting). **Clara J. Rodriguez‐Zarate:** Writing‐review & editing (supporting). **Frank V. Paladino:** Project administration (equal); Supervision (equal); Writing‐original draft (supporting); Writing‐review & editing (supporting). **Mark Jordan:** Conceptualization (supporting); Methodology (supporting); Project administration (supporting); Supervision (equal); Writing‐original draft (supporting); Writing‐review & editing (supporting).

## Supporting information

Appendix S1

## Data Availability

The data available in this study are obtained from DNA sequences (GenBank Accession nos. MK749418‐MK749421) and microsatellite genotypes deposited on Dryad repository (https://doi.org/10.5061/dryad.c866t1g4f).

## References

[ece36564-bib-0001] Abreu‐Grobois, A. , & Plotkin, P. T. (2008). Lepidochelys olivacea. The IUCN Red List of Threatened Species, 2008, 6–8. 10.2305/IUCN.UK.2008.RLTS.T11534A3292503.en

[ece36564-bib-0002] Abreu‐Grobois, F. A. , Horrocks, J. A. , Formia, A. , Dutton, P. H. , LeRoux, R. , Velez‐Zuazo, X. , … Meylan, P. (2006). New mtDNA D‐loop primers which work for a variety of marine turtle species may increase the resolution of mixed stock analysis. In *Proceedings of the 26th Annual Symposium on Sea Turtle Biology*, Book of Abstracts, p. 179.

[ece36564-bib-0003] Aggarwal, R. K. , Lalremruata, A. , Velavan, T. P. , Pavani Sowjanya, A. , & Singh, L. (2008). Development and characterization of ten novel microsatellite markers from olive ridley sea turtle (*Lepidochelys olivacea*). Conservation Genetics, 9(4), 981–984. 10.1007/s10592-007-9421-0

[ece36564-bib-0004] Aggarwal, R. K. , Velavan, T. P. , Udaykumar, D. , Hendre, P. S. , Shanker, K. , Choudhury, B. C. , & Singh, L. (2004). Development and characterization of novel microsatellite markers from the olive ridley sea turtle (*Lepidochelys olivacea*). Molecular Ecology Notes, 4(1), 77–79. 10.1046/j.1471-8286.2003.00574.x

[ece36564-bib-0005] Avise, J. C. , Bowen, B. W. , Lamb, T. , Meylan, A. B. , & Bermingham, E. (1992). Mitochondrial DNA evolution at a turtle’s pace: evidence for low genetic variability and reduced microevolutionary rate in the Testudines. Molecular Biology and Evolution, 9(3), 457–473. 10.1093/oxfordjournals.molbev.a040735 1584014

[ece36564-bib-0006] Bahri, S. , Atmadipoera, A. S. , & Madduppa, H. H. (2018). Genetic diversity of olive ridley Lepidochelys olivacea associated with current pattern in Cendrawasih Bay, Papua. Jurnal Ilmu Dan Teknologi Kelautan Tropis, 9(2), 747 10.29244/jitkt.v9i2.19307

[ece36564-bib-0007] Bernardo, J. , & Plotkin, P. T. (2007). An evolutionary perspective on the arribada phenomenon and reproductive behavioral polymorphism of olive ridley sea turtles (*Lepidochelys olivacea*). The Biology and Conservation of Ridley Sea Turtles, 1, 58–87.

[ece36564-bib-0008] Blouin, M. S. (2003). DNA‐based methods for pedigree reconstruction and kinship analysis in natural populations. Trends in Ecology & Evolution, 18(10), 503–511. 10.1016/S0169-5347(03)00225-8

[ece36564-bib-0009] Bowen, B. W. , Clark, A. M. , Abreu‐Grobois, F. A. , Chaves, A. , Reichart, H. A. , & Ferl, R. J. (1997). Global phylogeography of the ridley sea turtles (*Lepidochelys* spp.) as inferred from mitochondrial DNA sequences. Genetica, 101(3), 179–189. 10.1023/A:1018382415005 9692227

[ece36564-bib-0010] Bowen, B. W. , & Karl, S. A. (2007). Population genetics and phylogeography of sea turtles. Molecular Ecology, 16(23), 4886–4907. 10.1111/j.1365-294X.2007.03542.x 17944856

[ece36564-bib-0011] Campista León, S. , Beltrán Espinoza, J. A. , Sosa Cornejo, I. , Castillo Ureta, H. , Martín del CampoFlores, J. R. , Sánchez Zazueta, J. G. , & Peinado Guevara, L. I. (2019). ‘Haplotypic characterization of the olive ridley turtle (*Lepidochelys olivacea*) in northwest Mexico: The northernmost limit of its distribution. Animal Biodiversity and Conservation, 42(1), 113–126. 10.32800/abc.2019.42.0113

[ece36564-bib-0084] Carreras, C. , Pascual, M. , Cardona, L. , Aguilar, A. , Margaritoulis, D. , Rees, A. , … Khalil, M. (2007). The genetic structure of the loggerhead sea turtle (Caretta caretta) in the Mediterranean as revealed by nuclear and mitochondrial DNA and its conservation implications. Conservation Genetics, 8, 761–775.

[ece36564-bib-0012] Clement, M. , Posada, D. , & Crandall, K. A. (2000). TCS: A computer program to estimate gene genealogies. Molecular Ecology, 9(10), 1657–1659. 10.1046/j.1365-294X.2000.01020.x 11050560

[ece36564-bib-0013] Cornuet, J. M. , & Luikart, G. (1996). Description and power analysis of two tests for detecting recent population bottlenecks from allele frequency data. Genetics, 144(4), 2001–2014. 10.1093/oxfordjournals.jhered.a111627 8978083 PMC1207747

[ece36564-bib-0014] Dapp, D. , Arauz, R. , Spotila, J. R. , & O'Connor, M. P. (2013). ‘Impact of Costa Rican longline fishery on its bycatch of sharks, stingrays, bony fish and olive ridley turtles (*Lepidochelys olivacea*). Journal of Experimental Marine Biology and Ecology, 448, 228–239. 10.1016/j.jembe.2013.07.014

[ece36564-bib-0085] Dewald, J. R. , & Pike, D. A. (2014). Geographical variation in hurricane impacts among sea turtle populations. Journal of Biogeography, 41, 307–316.

[ece36564-bib-0115] Di Rienzo, A. , Peterson, A. C. , Garza, J. C. , Valdes, A. M. , Slatkin, M. , & Freimer, N. B. (1994). Mutational processes of simplesequence repeat loci in human populations. Proceedings of the National Academy of Sciences, 91, 3166–3170. 10.1073/pnas.91.8.3166 PMC43536 8159720

[ece36564-bib-0015] Dornfeld, T. C. , Robinson, N. J. , Tomillo, P. S. , & Paladino, F. V. (2015). Ecology of solitary nesting olive ridley sea turtles at Playa Grande, Costa Rica. Marine Biology, 162(1), 123–139. 10.1007/s00227-014-2583-7

[ece36564-bib-0016] Dutton, P. H. , & Stewart, K. R. (2013). A Method for Sampling Hatchling Sea Turtles for the Development of a Genetic Tag. Marine Turtle Newsletter, 138, 3–7.

[ece36564-bib-0017] Eguchi, T. , Gerrodette, T. , Pitman, R. L. , Seminoff, J. A. , & Dutton, P. H. (2007). At‐sea density and abundance estimates of the olive ridley turtle *Lepidochelys olivacea* in the eastern tropical Pacific. Endangered Species Research, 3(2), 191–203. 10.3354/esr003191

[ece36564-bib-0018] Evanno, G. , Regnaut, S. , & Goudet, J. (2005). Detecting the number of clusters of individuals using the software STRUCTURE: A simulation study. Molecular Ecology, 14(8), 2611–2620. 10.1111/j.1365-294X.2005.02553.x 15969739

[ece36564-bib-0019] Excoffier, L. , & Lischer, H. E. L. (2010). Arlequin suite ver 3.5: A new series of programs to perform population genetics analyses under Linux and Windows. Molecular Ecology Resources, 10(3), 564–567. 10.1111/j.1755-0998.2010.02847.x 21565059

[ece36564-bib-0020] Falush, D. , Stephens, M. , & Pritchard, J. K. (2007). Inference of population structure using multilocus genotype data: Dominant markers and null alleles. Molecular Ecology Notes, 7(4), 574–578. 10.1111/j.1471-8286.2007.01758.x 18784791 PMC1974779

[ece36564-bib-0021] Fonseca, G. L. A. , & Valverde, R. (2010). Reporte final de la anidación de tortuga lora (*Lepidochelys olivacea*), Playa Nancite, Parque Nacional Santa Rosa, Costa Rica. Available at: http://copa.acguanacaste.ac.cr/handle/11606/636 Accessed: 23 March 2020.

[ece36564-bib-0022] Fonseca, L. G. , Murillo, G. A. , Guadamúz, L. , Spínola, R. M. , & Valverde, R. A. (2009). Downward but Stable Trend in the Abundance of Arribada Olive Ridley Sea Turtles (*Lepidochelys olivacea*) at Nancite Beach, Costa Rica (1971–2007). Chelonian Conservation and Biology Journal, 8(1), 19–27. 10.2744/ccb-0739.1

[ece36564-bib-0023] Grafen, A. (1985). A geometric view of relatedness. Oxford Surveys in Evolutionary Biology, 2, 28–89.

[ece36564-bib-0024] Hahn, Anelise Torres , Naro‐Maciel, Eugenia , Jensen, Michael , Bowen, Brian , Comin de Castilhos, Jaqueline , Abreu‐Grobois, Alberto , & de Thoisy, Benoit . (2013). Phylogeography of olive ridley turtles In Tucker T., Belskis L,, Panagopoulou A,, Rees A,, Frick M., Williams K., & Stewart K. (Eds.), 33rd ISTS Symposium on Sea Turtle Biology and Conservation (p. 226). Silver Spring, MD: National Oceanographic and Atmospheric Administration https://pdfs.semanticscholar.org/8f90/b6f8b2aead2aa2c2f3915045

[ece36564-bib-0025] Hamann, M. , Godfrey, M. H. , Seminoff, J. A. , Arthur, K. , Barata, P. , Bjorndal, K. A. , … Godley, B. J. (2010). Global research priorities for sea turtles: Informing management and conservation in the 21st century. Endangered Species Research, 11(3), 245–269. 10.3354/esr00279

[ece36564-bib-0027] Honarvar, S. , Spotila, J. R. , & O’Connor, M. P. (2011). Microbial community structure in sand on two olive ridley arribada nesting beaches, Playa La Flor, Nicaragua and Playa Nancite, Costa Rica. Journal of Experimental Marine Biology and Ecology, 409(1‐2), 339–344. 10.1016/j.jembe.2011.09.015

[ece36564-bib-0028] Hope, R. A. (2002). Wildlife harvesting, conservation and poverty: The economics of olive ridley egg exploitation. Environmental Conservation, 29(3), 375–384. 10.1017/S0376892902000255

[ece36564-bib-0030] Jensen, M. P. , Abreu‐grobois, F. A. , Frydenberg, J. , & Loeschcke, V. (2006). Microsatellites provide insight into contrasting mating patterns in arribada vs. non‐arribada olive ridley sea turtle rookeries. Molecular Ecology, 15(9), 2567–2575. 10.1111/j.1365-294X.2006.02951.x 16842427

[ece36564-bib-0031] Jensen, M. P. , Limpus, C. J. , Whiting, S. D. , Guinea, M. , Prince, R. , Dethmers, K. , … FitzSimmons, N. N. (2013). Defining olive ridley turtle *Lepidochelys olivacea* management units in Australia and assessing the potential impact of mortality in ghost nets. Endangered Species Research, 21(3), 241–253. 10.3354/esr00521

[ece36564-bib-0032] Jombart, T. (2008). Adegenet: A R package for the multivariate analysis of genetic markers. Bioinformatics, 24(11), 1403–1405. 10.1093/bioinformatics/btn129 18397895

[ece36564-bib-0033] Jombart, T. , Devillard, S. , & Balloux, F. (2010). Discriminant analysis of principal components: A new method for the analysis of genetically structured populations. BMC Genetics, 11(1), 94 10.1186/1471-2156-11-94 20950446 PMC2973851

[ece36564-bib-0034] Jones, O. R. , & Wang, J. (2010). COLONY: a program for parentage and sibship inference from multilocus genotype data. Molecular Ecology Resources, 10(3), 551–555. 10.1111/j.1755-0998.2009.02787.x 21565056

[ece36564-bib-0035] Jost, L. (2008). GST and its relatives do not measure differentiation. Molecular Ecology, 17(18), 4015–4026. 10.1111/j.1365-294X.2008.03887.x 19238703

[ece36564-bib-0036] Kalb, H. (1999). Behavior and physiology of solitary and arribada nesting Olive Ridley sea turtles (*Lepidochelys olivacea*) during the internesting period. College Station, TX: Texas A and M University.

[ece36564-bib-0037] Kalinowski, S. T. (2011). The computer program STRUCTURE does not reliably identify the main genetic clusters within species: Simulations and implications for human population structure. Heredity, 106(4), 625–632. 10.1038/hdy.2010.95 20683484 PMC3183908

[ece36564-bib-0038] Kearse, M. , Moir, R. , Wilson, A. , Stones‐Havas, S. , Cheung, M. , Sturrock, S. , … Drummond, A. (2012). Geneious Basic: An integrated and extendable desktop software platform for the organization and analysis of sequence data. Bioinformatics, 28(12), 1647–1649. 10.1093/bioinformatics/bts199 22543367 PMC3371832

[ece36564-bib-0039] Komoroske, L. M. , Jensen, M. P. , Stewart, K. R. , Shamblin, B. M. , & Dutton, P. H. (2017). Advances in the Application of Genetics in Marine Turtle Biology and Conservation. Frontiers in Marine Science, 4, 156 10.3389/fmars.2017.00156

[ece36564-bib-0140] Lee, P. L. , Schofield, G. , Haughey, R. I. , Mazaris, A. D. , & Hays, G. C. (2018). A review of patterns of multiple paternity across sea turtle rookeries. Advances in Marine Biology, 79, 1–31. 30012274 10.1016/bs.amb.2017.09.004

[ece36564-bib-0040] Li, Y. L. , & Liu, J. X. (2018). StructureSelector: A web‐based software to select and visualize the optimal number of clusters using multiple methods. Molecular Ecology Resources, 18(1), 176–177. 10.1111/1755-0998.12719 28921901

[ece36564-bib-0041] Lohmann, K. J. , Putman, N. F. , & Lohmann, C. M. F. (2008). Geomagnetic imprinting: A unifying hypothesis of long‐distance natal homing in salmon and sea turtles. National Acadamic Sciences, Retrieved from www.pnas.orgcgidoi10.1073pnas.0801859105 10.1073/pnas.0801859105 PMC2614721 19060188

[ece36564-bib-0042] Lohmann, K. J. , Witherington, B. E. , Lohmann, C. M. F. , & Salmon, M. (2017) Orientation, navigation, and natal beach homing in sea turtles. The Biology of Sea Turtles, 1, 107–135. 10.1201/9780203737088

[ece36564-bib-0043] López‐Castro, M. C. , & Rocha‐Olivares, A. (2005). The panmixia paradigm of eastern Pacific olive ridley turtles revised: Consequences for their conservation and evolutionary biology. Molecular Ecology, 14(11), 3325–3334. 10.1111/j.1365-294X.2005.02652.x 16156806

[ece36564-bib-0144] Luikart, G. , Allendorf, F. W. , Cornuet, J. M. , & Sherwin, W. B. (1998). Distortion of allele frequency distributions provides a test for recent population bottlenecks. Journal of Heredity, 89, 238–247. 9656466 10.1093/jhered/89.3.238

[ece36564-bib-0044] Lynch, M. , & Ritland, K. (1999) Estimation of pairwise relatedness with molecular markers. Genetics, 152(4), 1753–1766. 10430599 10.1093/genetics/152.4.1753 PMC1460714

[ece36564-bib-0045] Mansfield, K. L. , Wyneken, J. , Porter, W. P. , & Luo, J. (2014). First satellite tracks of neonate sea turtles redefine the ‘lost years’ oceanic niche. Proceedings of the Royal Society B: Biological Sciences, 281(1781), 20133039. 10.1098/rspb.2013.3039 PMC3953841 24598420

[ece36564-bib-0046] Márquez, M. R. , Peñaflores, C. , & Vasconcelos, J. C. (1996) Olive ridley turtles (*Lepidochelys olivacea*) show signs of recovery at La Escobilla, Oaxaca. Marine Turtle Newsletter, 73, 5–7.

[ece36564-bib-0047] Montero, J. T. , Rincon, R. O. M. , Heppell, S. S. , & Hall, M. A. (2016). Characterizing environmental and spatial variables associated with the incidental catch of olive ridley (*Lepidochelys olivacea*) in the Eastern Tropical Pacific purse‐seine fishery. Fisheries Oceanography, 25(1), 1–14. 10.1111/fog.12130

[ece36564-bib-0048] Moore, J. E. , Wallace, B. P. , Lewison, R. L. , Žydelis, R. , Cox, T. M. , & Crowder, L. B. (2009). A review of marine mammal, sea turtle and seabird bycatch in USA fisheries and the role of policy in shaping management. Marine Policy, 33(3), 435–451. 10.1016/j.marpol.2008.09.003

[ece36564-bib-0049] Múrias dos Santos, A. , Cabezas, M. P. , Tavares, A. I. , Xavier, R. , & Branco, M. (2016). TcsBU: A tool to extend TCS network layout and visualization. Bioinformatics, 32(4), 627–628. 10.1093/bioinformatics/btv636 26515821

[ece36564-bib-0050] Ohta, T. , & Kimura, M. (2008). A model of mutation appropriate to estimate the number of electrophoretically detectable alleles in a finite population. Genetics Research, 89(5–6), 367–370. 10.1017/S0016672308009531 18976523

[ece36564-bib-0051] Peakall, R. , & Smouse, P. E. (2006). GENALEX 6: Genetic analysis in Excel. Population genetic software for teaching and research. Molecular Ecology Notes, 6(1), 288–295. 10.1111/j.1471-8286.2005.01155.x PMC3463245 22820204

[ece36564-bib-0052] Peery, M. Z. , Kirby, R. , Reid, B. N. , Stoelting, R. , Doucet‐bëer, E. , Robinson, S. , … Palsbøll, P. J. (2012). Reliability of genetic bottleneck tests for detecting recent population declines. Molecular Ecology, 21(14), 3403–3418. 10.1111/j.1365-294X.2012.05635.x 22646281

[ece36564-bib-0053] Perrin, N. , & Goudet, J. (2001) Inbreeding, kinship, and the evolution of natal dispersal Clobert J. Dispersal, 123–142). Oxford, United Kingdom: Oxford University Press.

[ece36564-bib-0055] Piry, S. , Luikart, G. , & Cornuet, J. M. (1999). BOTTLENECK: A computer program for detecting recent reductions in the effective population size using allele frequency data. Journal of Heredity, 90(4), 502–503. 10.1093/jhered/90.4.502

[ece36564-bib-0056] Plot, V. , de Thoisy, B. , Blanc, S. , Kelle, L. , Lavergne, A. , Roger‐Bérubet, H. , … Georges, J.‐Y. (2012). Reproductive synchrony in a recovering bottlenecked sea turtle population. Journal of Animal Ecology, 81(2), 341–351. 10.1111/j.1365-2656.2011.01915.x 22007680

[ece36564-bib-0057] Plotkin, P. T. (2010). Nomadic behaviour of the highly migratory olive ridley sea turtle Lepidochelys olivacea in the eastern tropical Pacific Ocean. Endangered Species Research, 13(1), 33–40. 10.3354/esr00314

[ece36564-bib-0160] Pritchard, J. , Stephens, M. , & Donnelly, P. (2000). Inference of population structure using multilocus genotype data. Genetics Society of America, 155(3), 945–959. 10.1093/genetics/155.2.945 PMC1461096 10835412

[ece36564-bib-0060] Puechmaille, S. J. (2016). The program structure does not reliably recover the correct population structure when sampling is uneven: Subsampling and new estimators alleviate the problem. Molecular Ecology Resources, 16(3), 608–627. 10.1111/1755-0998.12512 26856252

[ece36564-bib-0061] Putman, A. I. , & Carbone, I. (2014). Challenges in analysis and interpretation of microsatellite data for population genetic studies. Ecology and Evolution, 4(22), 4399–4428. 10.1002/ece3.1305 25540699 PMC4267876

[ece36564-bib-0062] Queller, D. C. , & Goodnight, K. F. (1989). Estimating relatedness using genetic markers. Evolution, 43(2), 258–275. 10.1111/j.1558-5646.1989.tb04226.x 28568555

[ece36564-bib-0063] Rees, A. F. , Alfaro‐Shigueto, J. , Barata, P. , Bjorndal, K. A. , Bolten, A. B. , Bourjea, J. , … Godley, B. J. (2016). Are we working towards global research priorities for management and conservation of sea turtles? Endangered Species Research, 31(1), 337–382. 10.3354/esr00801

[ece36564-bib-0064] Reid, B. N. , Thiel, R. P. , Palsbøll, P. J. , & Peery, M. Z. (2016). Linking genetic kinship and demographic analyses to characterize dispersal: Methods and application to blanding’s turtle. Journal of Heredity, 107(7), 603–614. 10.1093/jhered/esw052 27552818

[ece36564-bib-0065] Revuelta, O. et al (2015). First report of an olive ridley (*Lepidochelys olivacea*) in the Mediterranean Sea. Mediterranean Marine Science, 16(2), 346–351. 10.12681/mms.1131

[ece36564-bib-0066] Rice, W. R. (1989). Analyzing tables of statistical tests. Evolution, 43(1), 223–225. 10.1111/j.1558-5646.1989.tb04220.x 28568501

[ece36564-bib-0067] Roberts, M. A. , Schwartz, T. S. , & Karl, S. A. (2004). Global Population Genetic Structure and Male‐Mediated Gene Flow in the Green Sea Turtle (*Chelonia mydas*): Analysis of Microsatellite Loci. Genetics, 166(4), 1857–1870. 10.1534/genetics.166.4.1857 15126404 PMC1470815

[ece36564-bib-0068] Rodríguez‐Zárate, C. J. , Rocha‐Olivares, A. , & Beheregaray, L. B. (2013). Genetic signature of a recent metapopulation bottleneck in the olive ridley turtle (*Lepidochelys olivacea*) after intensive commercial exploitation in Mexico. Biological Conservation, 168, 10–18. 10.1016/j.biocon.2013.09.009

[ece36564-bib-0069] Rodríguez‐Zárate, C. J. , Sandoval‐Castillo, J. , van Sebille, E. , Keane, R. G. , Rocha‐Olivares, A. , Urteaga, J. , & Beheregaray, L. B. (2018) Isolation by environment in the highly mobile olive ridley turtle (*Lepidochelys olivacea*) in the eastern pacific. Proceedings of the Royal Society B: Biological Sciences. Royal Society Publishing, 285(1878), 20180264 10.1098/rspb.2018.0264. PMC5966603 29720414

[ece36564-bib-0070] Ronce, O. , Gandon, S. , & Rousset, F. (2000). Kin selection and natal dispersal in an age‐structured population. Theoretical Population Biology, 58(2), 143–159. 10.1006/tpbi.2000.1476 11042105

[ece36564-bib-0071] Rousset, F. (2008). GENEPOP’007: A complete re‐implementation of the GENEPOP software for Windows and Linux. Molecular Ecology Resources, 8(1), 103–106. 10.1111/j.1471-8286.2007.01931.x 21585727

[ece36564-bib-0072] Rousset, F. , & Raymond, M. (1995). Testing heterozygote excess and deficiency. Genetics, 140(4), 1413–1419. 7498780 10.1093/genetics/140.4.1413 PMC1206704

[ece36564-bib-0073] Rozas, J. , Ferrer‐Mata, A. , Sánchez‐DelBarrio, J. C. , Guirao‐Rico, S. , Librado, P. , Ramos‐Onsins, S. E. , & Sánchez‐Gracia, A. (2017). DnaSP 6: DNA sequence polymorphism analysis of large data sets. Molecular Biology and Evolution, 34(12), 3299–3302. 10.1093/molbev/msx248 29029172

[ece36564-bib-0074] Sainudiin, R. , Durrett, R. T. , Aquadro, C. F. , & Nielsen, R. (2004). Microsatellite mutation models: Insights from a comparison of humans and chimpanzees. Genetics, 168(1), 383–395. 10.1534/genetics.103.022665 15454551 PMC1448085

[ece36564-bib-0075] Schunter, C. , Pascual, M. , Garza, J. C. , Raventos, N. , & Macpherson, E. (2014). Kinship analyses identify fish dispersal events on a temperate coastline. Proceedings of the Royal Society B: Biological Sciences, 281(1785), 20140556. 10.1098/rspb.2014.0556 PMC4024307 24812064

[ece36564-bib-0077] Shanker, K. , Ramadevi, J. , Choudhury, B. C. , Singh, L. , & Aggarwal, R. K. (2004). Phylogeography of olive ridley turtles (*Lepidochelys olivacea*) on the east coast of India: Implications for conservation theory. Molecular Ecology, 13(7), 1899–1909. 10.1111/j.1365-294X.2004.02195.x 15189212

[ece36564-bib-0078] Spotila, J. (2004). Sea turtles: A complete guide to their biology, behavior, and conservation. Johns Hopkins University Press https://books.google.com/books?hl=en&lr=&id=dpsJrFxVIvUC&oi=fnd&pg=PP15&dq=spotilla+biology+of+sea+turtles&ots=t7alaI9Iqc&sig=DhZHJJT984gdLnFeTmMvyfSQ_40

[ece36564-bib-0079] Thompson, J. D. , Gibson, T. J. , & Higgins, D. G. (2003). Multiple sequence alignment using ClustalW and ClustalX. Current Protocols in Bioinformatics, 00(1), 2.3.1–2.3.22. 10.1002/0471250953.bi0203s00 18792934

[ece36564-bib-0080] Trullas, S. C. , Spotila, J. R. , & Paladino, F. V. (2006). Energetics during hatchling dispersal of the olive ridley turtle *Lepidochelys olivacea* using doubly labeled water. Physiological and Biochemical Zoology, 79(2), 389–399. 10.1086/499982 16555197

[ece36564-bib-0081] Valverde, R. A. , Orrego, C. M. , Tordoir, M. T. , Gómez, F. M. , Solís, D. S. , Hernández, R. A. , … Spotila, J. R. (2012). Olive Ridley Mass Nesting Ecology and Egg Harvest at Ostional Beach, Costa Rica. Chelonian Conservation and Biology Journal, 11(1), 1–11. 10.2744/ccb-0959.1

[ece36564-bib-0082] Weir, B. S. , & Cockerham, C. C. (1984). Estimating F‐statistics for the analysis of population structure. Evolution, 38(6), 1358–1370. 10.1111/j.1558-5646.1984.tb05657.x 28563791

[ece36564-bib-0083] Williamson‐Natesan, E. G. (2005). Comparison of methods for detecting bottlenecks from microsatellite loci. Conservation Genetics, 6(4), 551–562. 10.1007/s10592-005-9009-5

[ece36564-bib-0086] Wright, S. (1951). The genetical structure of populations. Annals of Eugenics, 15, 323–354. 24540312 10.1111/j.1469-1809.1949.tb02451.x

