# Supplementary material for: Large‐scale connectivity, cryptic population structure, and relatedness in Eastern Pacific Olive ridley sea turtles (Lepidochelys olivacea)
Source: Ecol Evol. 2020 Jul 19;10(16):8688–704. doi: 10.1002/ece3.6564 (PMC7452818; doi:10.1002/ece3.6564)
Supplement: Supplementary file 1 — Appendix S1 [file ECE3-10-8688-s001.docx]

**Appendix**

Figures


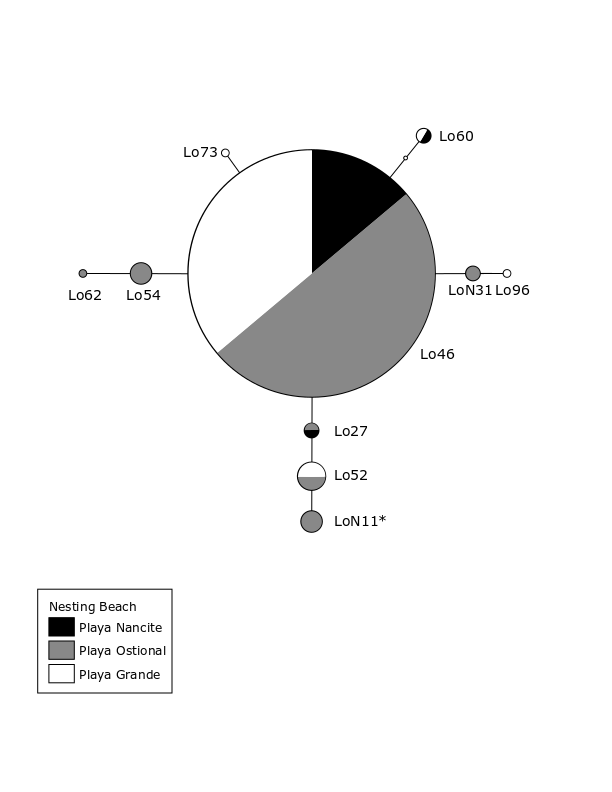


Figure 1. Network of olive ridley mtCR haplotypes found at three sites in Costa Rica. Node size corresponds to relative haplotype frequency. Bars in between nodes represent one mutational step. Nodes without names are mutational steps not reported from this study.


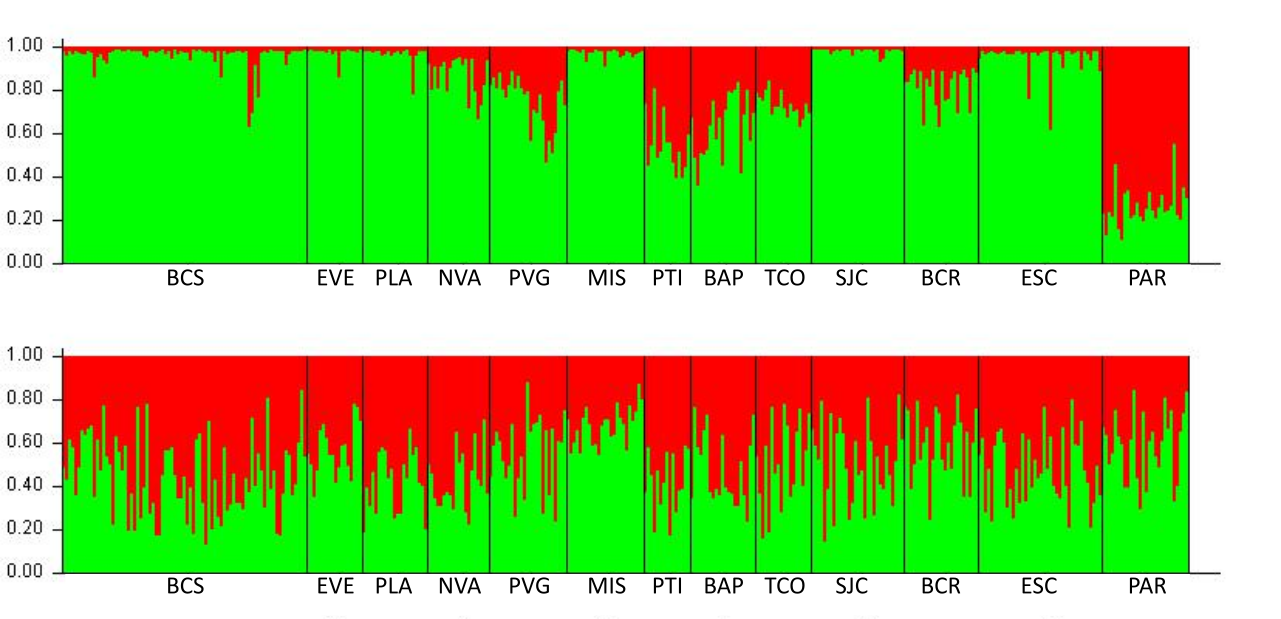


Figure 2. STRUCTURE bar plots from Mexican olive ridleys. Colors correspond to different populations, and bar heights correspond to the probability of an individual being assigned to each population. STRUCTURE was run with (top) and without (bottom) location as a prior. Abbreviations correspond to sampling sites as published in Rodriguez-Zarate et al. (2018; Table 1)

Tables

| **Locus** | **Primer Sequence (5’-3’)** | **Repeat Motif** | **Temp (°C)** | **Size Range (bp)** | **N** | ***k*** | ***Ne*** | ***Ho*** | ***He*** |
| --- | --- | --- | --- | --- | --- | --- | --- | --- | --- |
| OR2 | F: GCTCCTGCATCACTATTTCCTGTT | (GT)_8_GCC(GT)_5_ | 61 | 166-198 (166) | 114 | 12 | 4.124 | 0.895 | 0.758 |
|  | R: CAGTCGGGCGTCATCATGCTGCCCCCACACCCTCG |  |  |  |  |  |  |  |  |
| OR4 | F: AGGCACACTAACAGAGAACTTGG | (TG)_9_...(TG)_23_ | 61 | 134-170 (144) | 117 | 19 | 11.474 | 0.957 | 0.913 |
|  | R: CAGTCGGGCGTCATCAGGGACCCTAAAATACCACAAGACA |  |  |  |  |  |  |  |  |
| OR7 | F: GGGTTAGATATAGGAGGTGCTTGATGT | (GT)_6_(GA)_7_ | 61 | 202-228(206) | 118 | 12 | 5.154 | 0.932 | 0.806 |
|  | R: CAGTCGGGCGTCATCATCAGGATTAGCCAACAAGAGCAAAA |  |  |  |  |  |  |  |  |
| OR9 | F: GCCCCCACCGAGGACAAAAG | (GA)_16_ | 61 | 173-185 (179) | 116 | 7 | 2.746 | 0.922 | 0.636 |
|  | R: CAGTCGGGCGTCATCATTTTCACTCAACCTGTAATCCACCTCAT |  |  |  |  |  |  |  |  |
| OR11 | F: TGAGCACTGCAAATGGAGGATGGT | (GA)_22_ | 65* | 215-257 (245) | 110 | 19 | 11.07 | 0.882 | 0.91 |
|  | R: CAGTCGGGCGTCATCAAGTGCCTGATTCTTCGAGTTGCTGAG |  |  |  |  |  |  |  |  |
| OR16 | F: CAAGGTTAGATATAGGAGGTGCTGATGT | (GT)_5_(GA)_7_ | 61 | 237-263 (241) | 108 | 12 | 4.396 | 0.75 | 0.773 |
|  | R: CAGTCGGGCGTCATCACCTGCTTTGAATCCTGCCATAGTAATC |  |  |  |  |  |  |  |  |
| OR18 | F: AAACACCAGAATAGAGGCTCAAACT | (AC)_11_ | 61 | 131-149 (139) | 118 | 6 | 2.051 | 0.602 | 0.512 |
|  | R: CAGTCGGGCGTCATCATCTCTGGGCTGCCTACTTTATTC |  |  |  |  |  |  |  |  |
| OR22 | F: AAGTCCTGTTGAATCCTGCCATAG | (CT)_7_(CA)_6_ | 61 | 237-265 (243) | 116 | 13 | 4.72 | 0.819 | 0.788 |
|  | R: CAGTCGGGCGTCATCAGGTTAGATATAGGAGGTGCTGATGTTA |  |  |  |  |  |  |  |  |

Table 1. Characteristics of eight microsatellite loci for 118 olive ridleys sampled from Costa Rica. OR2, OR4, and OR7 are from Aggarwal et al. (2004). OR9 through OR22 are from Aggarwal et al. (2008). OR7 and 22 were excluded from analyses due to linkage with OR16. Temp = annealing temperature used in PCR amplification. “*” indicates a touchdown of 1°C/cycle from 65°C to 530C. N = number of individuals genotyped; k = number of alleles; Ne = number of effective alleles; Ho = observed heterozygosity; He =expected heterozygosity.
